# Comparative Biofilm Assays Using Enterococcus faecalis OG1RF Identify New Determinants of Biofilm Formation

**DOI:** 10.1128/mBio.01011-21

**Published:** 2021-06-15

**Authors:** Julia L. E. Willett, Jennifer L. Dale, Lucy M. Kwiatkowski, Jennifer L. Powers, Michelle L. Korir, Rhea Kohli, Aaron M. T. Barnes, Gary M. Dunny

**Affiliations:** a Department of Microbiology and Immunology, University of Minnesota Medical School, Minneapolis, Minnesota, USA; University of Pittsburgh

**Keywords:** antibiotic resistance, biofilm infections, functional genomics, gene discovery

## Abstract

Enterococcus faecalis is a common commensal organism and a prolific nosocomial pathogen that causes biofilm-associated infections. Numerous E. faecalis OG1RF genes required for biofilm formation have been identified, but few studies have compared genetic determinants of biofilm formation and biofilm morphology across multiple conditions. Here, we cultured transposon (Tn) libraries in CDC biofilm reactors in two different media and used Tn sequencing (TnSeq) to identify core and accessory biofilm determinants, including many genes that are poorly characterized or annotated as hypothetical. Multiple secondary assays (96-well plates, submerged Aclar discs, and MultiRep biofilm reactors) were used to validate phenotypes of new biofilm determinants. We quantified biofilm cells and used fluorescence microscopy to visualize biofilms formed by six Tn mutants identified using TnSeq and found that disrupting these genes (OG1RF_10350, *prsA*, *tig*, OG1RF_10576, OG1RF_11288, and OG1RF_11456) leads to significant time- and medium-dependent changes in biofilm architecture. Structural predictions revealed potential roles in cell wall homeostasis for OG1RF_10350 and OG1RF_11288 and signaling for OG1RF_11456. Additionally, we identified growth medium-specific hallmarks of OG1RF biofilm morphology. This study demonstrates how E. faecalis biofilm architecture is modulated by growth medium and experimental conditions and identifies multiple new genetic determinants of biofilm formation.

## INTRODUCTION

Enterococcus faecalis is an early colonizer of the human gastrointestinal (GI) tract, where it remains as a minor component of the healthy microbiota in adults (1–3). It is also a prolific opportunistic pathogen that causes biofilm-associated infections, such as infected root canals, bacterial endocarditis, and prosthetic joint infections, and is frequently isolated from polymicrobial infection sites, such as the urinary tract, burns, and diabetic foot ulcers (4–9). The ability of E. faecalis to thrive as both a commensal and a pathogen is due in part to intrinsic and acquired antibiotic resistance mechanisms, including biofilm formation (10–13). Biofilm development occurs in both the pathogenic and nonpathogenic lifestyles of this organism, and recent high-resolution microscopic analysis of E. faecalis biofilms formed in the murine GI tract revealed small matrix-encapsulated microcolonies of biofilm cells spread across the epithelial surface (14). Biofilms formed *in vivo* morphologically resemble those grown *in vitro* (15, 16).

Numerous model systems have been developed to study biofilm formation *in vitro*, including widely used 96-well plate assays, CDC biofilm reactors (CBRs) for assessing biofilms under shear stress and continuous nutrient exchange, and microscopy-based methods that enable fine-scale evaluation of biofilm morphology and matrix properties over a range of time scales (17–19). However, gene expression patterns, biofilm architecture, and genetic determinants of biofilm formation can vary dramatically in biofilms cultured in different model systems, and we have demonstrated that E. faecalis biofilm development is influenced by growth medium and nutrient availability (14, 20, 21). Therefore, comparative studies can be useful for understanding how biofilm formation, development, and composition vary across conditions. Incorporation of diverse experimental systems for biofilm growth into the validation of genetic screens using transposon (Tn) libraries may enhance the power of such screens.

Previously, we described the generation of two sequence-defined collections of E. faecalis OG1RF Tn mutants termed SmarT (Sequence-defined *mariner* Technology) libraries due to the high level of genetic coverage (insertions in ∼70% of genes and intergenic regions [IGRs]) with a minimal number of Tn mutants (22). SmarT Tn sequencing (TnSeq) library 1 contains 6,829 mutants with mutations in genes and intergenic regions. SmarT TnSeq library 2 is a subset of library 1 and contains 1,948 mutants with Tn insertions in intergenic regions or uncharacterized or poorly characterized genes (22, 23). These Tn libraries have been used to identify OG1RF genes important for cholic acid resistance, biofilm formation and biofilm-associated antibiotic resistance in microtiter plates, response to phage infection, vaginal colonization, and augmentation of Escherichia coli growth (9, 22–27). However, to date, no studies have used E. faecalis Tn libraries for TnSeq studies to evaluate biofilm fitness determinants comprehensively.

Here, we used a variety of assays for analysis of genetic determinants of OG1RF biofilm formation *in vitro*. Using CBRs, we compared the biofilm fitness of OG1RF Tn mutants in multiple input libraries and in different growth media using TnSeq. We compared these results to previous genetic screens and identified a core set of OG1RF genes required for biofilm formation under multiple conditions. We then measured biofilm formation of a subset of Tn mutants in three secondary biofilm assays (microtiter plates, growth on submerged substrates, and miniature continuous-flow biofilm reactors). Additionally, we used bioinformatic tools to predict structure and function for poorly characterized biofilm determinants. Taken together, our data show that E. faecalis OG1RF encodes numerous previously unidentified determinants of biofilm formation, many of which affect biofilm architecture in a temporal and growth medium-dependent manner. Our primary and secondary screening approaches can also guide future studies of biofilm determinants and temporal morphology changes in other organisms.

## RESULTS

### Identification of biofilm determinants in E. faecalis using TnSeq.

**FIG 1:**
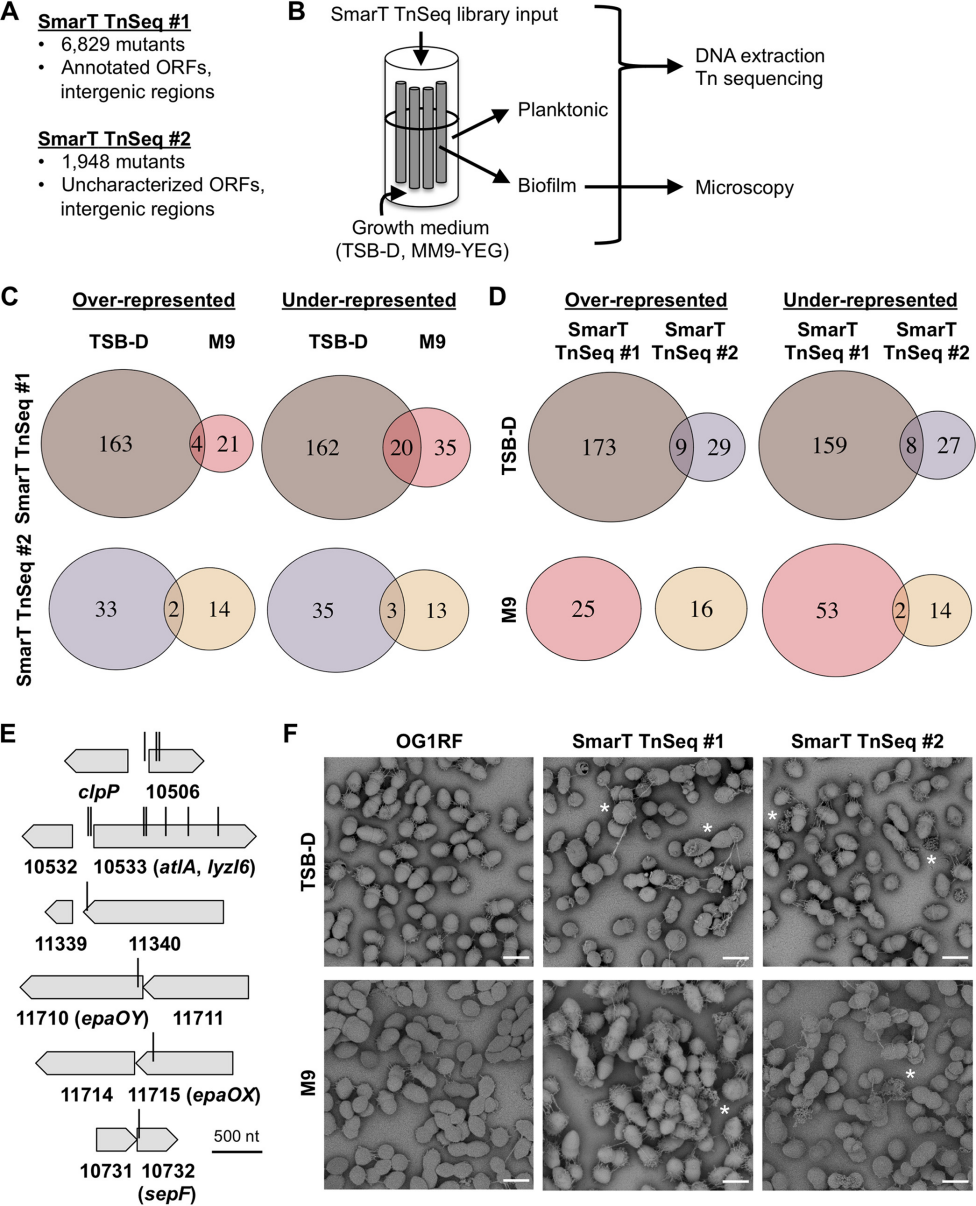
E. faecalis OG1RF biofilm formation in CDC reactors and summary of TnSeq. (A) Summary of SmarT TnSeq libraries used in this study. (B) Diagram showing CDC biofilm reactor (CBR) inoculation and sampling. (C) Venn diagrams summarizing differentially abundant (*P* < 0.05, no log_2_FC cutoff) Tn mutants from the same Tn library grown in different media. Venn diagrams in panels C and D were generated with the VennDiagram package for R. (D) Comparison of differentially abundant Tn mutants between the two SmarT TnSeq libraries grown in the same media. (E) Diagrams showing the most underrepresented Tn mutants from biofilm TnSeq. Vertical bars indicate Tn insertion sites. (F) Scanning electron microscopy images of biofilms from OG1RF and the SmarT TnSeq libraries cultured on Aclar membranes. Examples of misshapen cells and abundant extracellular material are marked with asterisks. Scale bars, 1 μm.

We sought to use the E. faecalis OG1RF SmarT libraries (Fig. 1A) to evaluate competitive fitness during biofilm formation in CDC biofilm reactors (CBRs) (22). We chose the CBRs for a primary biofilm screen because the system includes continuous flow and medium replacement and a relatively large surface area for biofilm development, decreasing the chance of “bottlenecking” and stochastic loss of mutants. OG1RF biofilms grown for 4 h in CBRs contain ∼10^6^ CFU/disc (28), so the colonization capacity of this niche is >1,000-fold greater than the number of mutants in our Tn libraries. The system also allows for direct, simultaneous comparison of the population distribution of mutants in the planktonic and biofilm states. We used each SmarT library to inoculate CBRs containing either tryptic soy broth without added dextrose (TSB-D) or modified M9 growth medium (MM9-YEG [29]) with ∼10^9^ CFU bacteria. Both media are routinely used to culture E. faecalis biofilms (16, 30). Cultures were grown with static incubation (4 to 6 h), after which a peristaltic pump was turned at a flow rate of 8 ml/min (18 to 20 h). DNA was isolated from input, planktonic, and biofilm samples, and Tn insertion sites were sequenced in order to determine the relative abundance of Tn mutants (Fig. 1B).

For each medium, we compared Tn abundance between planktonic and biofilm samples to identify mutants over- or underrepresented in biofilms, using a significance cutoff of *P* of <0.05 (Fig. 1C; see Table S1 in the supplemental material). We first examined Tn mutant abundance in SmarT TnSeq library 1. In TSB-D, 167 mutants were overrepresented and 182 mutants were underrepresented in biofilms relative to planktonic culture (Fig. S1A; Fig. 1C, brown circles). In MM9-YEG, 25 mutants were overrepresented and 55 mutants were underrepresented in biofilms (Fig. S1B; Fig. 1C, red circles). Four Tn mutants were overrepresented and 20 Tn mutants were underrepresented in both TSB-D and MM9-YEG biofilms.

10.1128/mBio.01011-21.1 FIG S1 Relative abundance of Tn mutants in CBR TnSeq and comparison with biofilm formation in microtiter plates. Panels A to D show data from CBR TnSeq, and panels E to H compare the fitness of mutants selected from the TnSeq to their phenotypes in monocultures using microtiter plate biofilm assays. (A to D) Volcano plots of SmarT TnSeq library 1 (6,829 mutants) in TSB-D (A) and MM9-YEG (B) and SmarT TnSeq library 2 (1,948 mutants) in TSB-D (C) and MM9-YEG (D). Tn mutants previously identified as biofilm determinants or chosen for microtiter plate assays are highlighted in purple. (E to H) Log_2_FC values from biofilm TnSeq were compared to biofilm index values obtained from microtiter plate biofilm assays for 6-h biofilms in TSB-D (E), 24-h biofilms in TSB-D (F), 6-h biofilms in MM9-YEG (G), and 24-h biofilms in MM9-YEG (H). For comparisons where Pearson’s coefficient values could be determined, they are shown in parentheses. Download FIG S1, TIF file, 0.3 MB. Copyright © 2021 Willett et al. 2021 Willett et al. https://creativecommons.org/licenses/by/4.0/ This content is distributed under the terms of the Creative Commons Attribution 4.0 International license.

10.1128/mBio.01011-21.8 TABLE S1 Tn mutants from SmarT TnSeq library 1 over- or underrepresented in biofilms relative to planktonic culture. Download Table S1, XLSX file, 0.1 MB. Copyright © 2021 Willett et al. 2021 Willett et al. https://creativecommons.org/licenses/by/4.0/ This content is distributed under the terms of the Creative Commons Attribution 4.0 International license.

A log_2_ fold change (log_2_FC) of ±1.5 was used as a cutoff to identify strongly underrepresented or overrepresented mutants. In TSB-D, 43 mutants had a log_2_FC of less than −1.5, and 3 had a log_2_FC of >1.5. In MM9-YEG, 20 mutants had a log_2_FC of less than −1.5, and 8 had a log_2_FC of >1.5 (Table S1; Fig. S1A and B). Notably, 13 mutants were strongly underrepresented in both media (Fig. 1E; Table 1). These include 2 with Tn insertions in OG1RF_10506, a hypothetical gene previously identified in a microtiter plate screen for biofilm-deficient mutants in TSB-D (23) and 5 with Tn insertions in *atlA* (OG1RF_10533, *lyzl6*), which encodes a major peptidoglycan hydrolase required for normal cell division and autolysis (31, 32). Additionally, a single Tn insertion in the intergenic region upstream of OG1RF_10506 (named Intergenic_535 based on sequential numbering of intergenic regions in the OG1RF genome) and 2 Tn insertions upstream of *atlA* (Intergenic_563) were underrepresented, suggesting that they could have polar effects on the transcription of OG1RF_10506 and *atlA*. Interestingly, Tn insertions in OG1RF_11710 (*epaOY* [25]) and OG1RF_11715 (*epaOX* [26]) were also strongly underrepresented in biofilms grown in both media. These genes are part of the locus encoding enterococcal polysaccharide antigen (*epa*) (33). Previous work from our laboratory has shown that *epa* genes are associated with biofilm-associated antibiotic resistance but that Tn insertions in *epa* genes did not lead to reduced biofilm formation in the absence of antibiotics in monoculture (26, 34).

**TABLE 1:** Tn mutants strongly underrepresented in biofilms grown in TSB-D and MM9-YEG

Locus tag	Nucleotide position	NCBI gene product description^*a*^	TSB-D		MM9-YEG	
			Log_2_FC	*P* value	Log_2_FC	*P* value
Intergenic_535	529929	NA	−3.23	4.18E−13	−1.56	6.01E−27
OG1RF_10506	530038	Hypothetical protein	−2.76	2.73E−51	−1.86	1.17E−78
OG1RF_10506	530068	Hypothetical protein	−2.79	3.60E−32	−1.77	7.09E−43
Intergenic_563	558300	NA	−2.69	1.53E−3	−1.62	2.11E−4
Intergenic_563	558335	NA	−3.03	7.64E−154	−1.93	8.62E−165
OG1RF_10533	559055	Cell wall lysis protein	−3.19	5.89E−159	−1.58	1.26E−102
OG1RF_10533	559075	Cell wall lysis protein	−3.26	1.72E−176	−1.79	2.49E−181
OG1RF_10533	559358	Cell wall lysis protein	−3.28	8.96E−35	−1.54	3.18E−31
OG1RF_10533	559660	Cell wall lysis protein	−3.19	6.76E−108	−2.22	1.44E−122
OG1RF_10533	560068	Cell wall lysis protein	−2.56	3.41E−279	−1.66	1.52E−112
OG1RF_11340	1403263	Acetaldehyde dehydrogenase	−2.96	1.87E−74	−1.79	2.17E−42
OG1RF_11710	1790332	O-antigen polymerase	−2.12	2.90E−4	−2.36	1.28E−14
OG1RF_11715	1794475	Glycosyltransferase	−3.93	9.82E−4	−4.84	1.87E−06

a NA, not applicable.

For SmarT TnSeq library 2, we again used a significance cutoff of *P* of <0.05 to identify Tn mutants differentially represented in biofilms compared to planktonic culture (Table S2; Fig. S1). In TSB-D, 35 mutants were overrepresented and 38 mutants were underrepresented in biofilms (Fig. S1C; Fig. 1C, purple circles). In MM9-YEG, 16 mutants were underrepresented and 16 mutants were overrepresented in biofilms (Fig. S1D; Fig. 1C, tan circles). Interestingly, we found relatively little overlap when comparing the two libraries in the same medium (Fig. 1D). In TSB-D, only 9 of 38 Tn mutants overrepresented in SmarT TnSeq library 2 were also overrepresented in SmarT TnSeq library 1, and only 8 of 35 Tn mutants underrepresented in SmarT TnSeq library 2 were also underrepresented in SmarT TnSeq library 1 (Fig. 1D, brown and purple circles). There was no overlap of overrepresented mutants in MM9-YEG, and only 2 mutants were underrepresented in both libraries. These results suggest that the community composition affected the relative fitness of Tn mutants in the CBR TnSeq experiments.

10.1128/mBio.01011-21.9 TABLE S2 Tn mutants from SmarT TnSeq library 2 over- or underrepresented in biofilms relative to planktonic culture. Download Table S2, XLSX file, 0.05 MB. Copyright © 2021 Willett et al. 2021 Willett et al. https://creativecommons.org/licenses/by/4.0/ This content is distributed under the terms of the Creative Commons Attribution 4.0 International license.

Only 4 mutants were underrepresented in SmarT TnSeq library 2 using a log_2_FC cutoff of −1.5, so we used a log_2_FC cutoff of ±1 to identify strongly under- or overrepresented mutants in this library (Table S2). In TSB-D, 8 mutants had a log_2_FC of less than −1, including those with insertions in OG1RF_10506, Intergenic_563, and *bph*, which was previously identified as a gene coding for a phosphatase required for surface attachment and biofilm formation (23). No mutants had a log_2_FC of >1. A Tn mutation in OG1RF_10732, which encodes a SepF homolog (35, 36), was strongly underrepresented in both media (Fig. 1E). In previous studies, strains with this Tn mutation had various defects in *in vitro* biofilm formation relative to OG1RF (27, 35), although the specific contribution of SepF to cell division during planktonic and biofilm growth has yet to be reported in E. faecalis. The other Tn insertion strongly underrepresented in MM9-YEG is located in Intergenic_1271, which is between OG1RF_11216 and OG1RF_11217. The Tn insertion downstream of Intergenic_1271 in OG1RF_11217 was not underrepresented in either medium, suggesting that Intergenic_1271 may encode a small RNA or peptide that is specifically important for biofilm formation in MM9-YEG.

We also compared biofilms formed by wild-type OG1RF versus SmarT TnSeq input pools on Aclar substrates using scanning electron microscopy (SEM). Altered biofilm morphology was previously observed in a small pool containing 11 OG1RF Tn mutants in a mouse GI model system (14), and disruption of some *epa* genes led to altered biofilm architecture (16, 34). Parental OG1RF biofilms were visible as a monolayer of cells, with strands of extracellular material present between cells (Fig. 1F, left panels). Few cells had aberrant shapes or morphologies. Biofilms formed by the SmarT TnSeq libraries contained markedly more misshapen cells and dysmorphic extracellular material than parental OG1RF biofilms (Fig. 1F, center and right panels), suggesting that some Tn insertions in the library disrupt genes involved in cell shape homeostasis or cell division. While additional research is needed to better understand individual determinants of biofilm architecture present in the SmarT TnSeq libraries, these results suggest that both libraries contain a substantial number of mutants with altered cell morphologies that can still form biofilms within complex communities.

### Determination of core and CBR-specific accessory biofilm determinants.

In previously reported genetic screens for biofilm determinants, OG1RF Tn mutants were grown as monocultures in microtiter plates (23, 27). This closed, static environment with no competing strains is substantially different from that of CBRs. To extend our understanding of environmental effects on E. faecalis biofilm formation, we sought to determine the overlap between mutants identified from microtiter plate screens and CBR TnSeq, which could constitute core OG1RF biofilm determinants. Because previous screens used TSB-D and not MM9-YEG, we examined the TSB-D TnSeq data from both library 1 and library 2 in this analysis but not the MM9-YEG data sets. Previously, a total of 204 insertions in 179 genes were associated with statistically reduced biofilm formation (23, 27). Only 35 Tn mutants were identified in both TnSeq and microtiter plate screens (Table 2), including the biofilm-associated phosphatase gene *bph*, autolysin gene *atlA*, stress response genes *hrcA* and *dnaK*, and the *ebp* pilus operon (23, 37–39).

**TABLE 2:** Core E. faecalis OG1RF biofilm determinants identified in TnSeq and microtiter plate biofilm screens

Locus tag	Nucleotide position	Gene name	Description
Intergenic_442	427629		IGR between OG1RF_10412 and OG1RF_10413
Intergenic_464	449894		IGR between OG1RF_10434 and OG1RF_10435
OG1RF_10435	450277, 450467	*bph*	Biofilm phosphatase
Intergenic_482	469369		IGR between OG1RF_10452 and OG1RF_10453
OG1RF_10506	530068, 530167, 530274		Hypothetical protein
Intergenic_563	558335		IGR between OG1RF_10532 and OG1RF_10533
OG1RF_10533	559075	*atlA* or *lyzl6*	Autolysin, LysM peptidoglycan-binding domain-containing protein
OG1RF_10717	741838	*ahrC* or *argR3*	Arginine repressor
OG1RF_10868	904848, 905256, 905964	*ebpR*	M-protein *trans*-acting positive regulator
Intergenic_918	906315		IGR between OG1RF_10868 and OG1RF_10869
OG1RF_10869	906894	*ebpA*	Endocarditis and biofilm-associated pilus tip protein EbpA
OG1RF_10870	909926, 910620, 911022	*ebpB*	Endocarditis and biofilm-associated pilus minor subunit EbpB
OG1RF_10871	911547, 912937	*ebpC*	Endocarditis and biofilm-associated pilus major subunit EbpC
OG1RF_10872	913633	*bps* or *srtC*	Ebp pilus assembly class C sortase
OG1RF_10889	928107	*lepB*	Signal peptidase I
Intergenic_1006	995480		IGR between OG1RF_10954 and OG1RF_10955
Intergenic_1127	1118301		IGR between OG1RF_11075 and OG1RF_11076
OG1RF_11076	1118585	*hrcA*	Heat-inducible transcriptional repressor HrcA
OG1RF_11078	1120304	*dnaK*	Molecular chaperone DnaK
Intergenic_1130	1121988		IGR between OG1RF_11078 and OG1RF_11079
OG1RF_11674	1746502		DUF1831 domain-containing protein
Intergenic_2022	2075283		IGR between OG1RF_11962 and OG1RF_11963
Intergenic_2295	2348175		IGR between OG1RF_12228 and OG1RF_12229
OG1RF_12447	2581857		DUF3298 domain-containing protein
OG1RF_12502	2644218		WxL domain-containing protein
Intergenic_2613	2692363		IGR between OG1RF_r10012 and OG1RF_12535, encodes OG1RF_RS13855
OG1RF_12540	2699893		DUF1129 domain-containing protein

Next, we asked which Tn mutants were underrepresented in biofilm TnSeq but did not have reduced biofilm formation in previous studies. These mutants could have biofilm defects in a community of Tn mutants but not monoculture, or they could be accessory biofilm determinants that are important under flow conditions. Using a log_2_FC cutoff of −1 for the TnSeq results, we identified 55 Tn mutations in 45 genes that were not found in previous studies (Table 3). These include multiple genes in the *epa* operon (OG1RF_11710 [*epaOY*], OG1RF_11714, OG1RF_11715 [*epaOX*], OG1RF_11716, and OG1RF_11722 [*epaQ*]), genes encoding predicted LCP family cell wall-modifying enzymes (OG1RF_10350, OG1RF_11288) and putative transcriptional regulators (OG1RF_12423 and OG1RF_12531), and genes annotated as hypothetical (OG1RF_10968 and OG1RF_11630).

**TABLE 3:** Biofilm determinants not previously identified in genetic screens^*a*^

Position	Locus tag	Gene product description	TSB-D			MM9-YEG		
			*P* value (BF/plank)	Log_2_FC (BF/plank)	SmarT library	*P* value (BF/plank)	Log_2_FC (BF/plank)	SmarT library
362782	OG1RF_10350	Transcriptional regulator	1.31E−07	−1.66	1			
440158	OG1RF_10423	Peptidyl-prolyl *cis-trans* isomerase	1.14E−11	−1.58	1			
468267	OG1RF_10452	Trigger factor				1.77E−66	−1.10	1
529585	OG1RF_10505	ATP-dependent Clp protease proteolytic subunit	9.29E−05	−2.47	1			
529929	Intergenic_535		4.18E−13	−3.23	1	6.01E−27	−1.56	1
604451	OG1RF_10576	ATP-dependent RNA helicase DeaD	5.94E−20	−2.48	1			
605468	OG1RF_10576	ATP-dependent RNA helicase DeaD	9.09E−11	−1.46	1			
658201	OG1RF_10621	Amino acid ABC superfamily ATP binding cassette transporter, membrane protein	2.68E−07	−1.08	1			
659044	OG1RF_10621	Amino acid ABC superfamily ATP binding cassette transporter, membrane protein	1.35E−09	−1.06	1			
737316	Intergenic_743		3.93E−07	−1.99	1			
741027	OG1RF_10716	Hemolysin A	1.96E−10	−1.17	1			
759278	OG1RF_10734	S4 domain-containing protein YlmH				1.47E−14	−1.37	1
759717	OG1RF_10734	S4 domain-containing protein YlmH				1.52E−16	−1.10	1
1009844	OG1RF_10968	Hypothetical protein	2.34E−37	−1.48	2			
1208294	Intergenic_1210					1.62E−02	−1.17	1
1213789	OG1RF_11160	Thioesterase	1.29E−10	−1.92	1			
1252773	OG1RF_11197	ABC superfamily ATP binding cassette transporter, membrane protein				8.96E−04	−1.23	1
1272332	Intergenic_1271					1.79E−03	−1.03	#2
1287696	OG1RF_11230	SacPA operon antiterminator				1.62E−03	−1.41	1
1345158	OG1RF_11288	Transcriptional regulator				3.44E−03	−1.04	1
1372168	OG1RF_11314	Catalase				1.42E−06	−1.32	1
1376818	OG1RF_11317	PTS family beta-glucosides porter, IIABC component				7.96E−03	−1.42	1
1383159	OG1RF_11322	Beta-glucosidase				4.06E−02	−1.39	1
1403263	OG1RF_11340	Acetaldehyde dehydrogenase	1.87E−74	−2.96	1	2.17E−42	−1.79	1
1407029	OG1RF_11344	Ethanolamine ammonia-lyase large subunit				1.73E−05	−1.45	1
1420208	OG1RF_11357	GTP-sensing transcriptional pleiotropic repressor CodY	8.20E−17	−2.28	1			
1458455	Intergenic_1452		7.92E−33	−1.12	1			
1515092	OG1RF_11453	Catabolite control protein A				3.64E−02	−1.60	1
1517672	OG1RF_11456	Methyl-accepting chemotaxis family protein				4.19E−17	−1.95	1
1525694	OG1RF_11465	Phosphate transport system regulatory protein PhoU	8.59E−05	−1.64	1			
1526148	OG1RF_11465	Phosphate transport system regulatory protein PhoU	1.85E−06	−1.92	1			
1526222	OG1RF_11465	Phosphate transport system regulatory protein PhoU	2.50E−13	−1.36	1			
1699911	OG1RF_11630	Hypothetical protein				1.68E−06	−1.22	1
1766576	OG1RF_11693	Cobalt (Co^2+^) ABC superfamily ATP binding cassette transporter, membrane protein				1.69E−02	−1.21	1
1789261	OG1RF_11710	O-antigen polymerase	9.56E−05	−1.50	1			
1790332	OG1RF_11710	O-antigen polymerase	2.90E−04	−2.12	1	1.28E−14	−2.36	1
1793746	OG1RF_11714	Group 2 glycosyl transferase				1.91E−38	−2.55	1
1794475	OG1RF_11715	glycosyltransferase	9.82E−04	−3.93	1	1.87E−06	−4.84	1
1795969	OG1RF_11716	Group 2 glycosyl transferase				9.53E−05	−1.71	1
1803231	OG1RF_11722	Hypothetical protein				2.01E−06	−1.37	1
1893517	OG1RF_11796	Phosphoribosylaminoimidazole carboxylase ATPase subunit PurK				8.09E−13	−2.04	1
1894091	OG1RF_11796	Phosphoribosylaminoimidazole carboxylase ATPase subunit PurK				5.64E−09	−1.03	1
1894392	OG1RF_11796	Phosphoribosylaminoimidazole carboxylase ATPase subunit PurK				4.10E−09	−1.33	1
2099505	OG1RF_11987	ATP synthase F1 sector gamma subunit	1.38E−21	−1.30	1			
2150973	OG1RF_12034	Phosphoglycerate mutase	2.86E−46	−2.92	1			
2244864	OG1RF_12122	Stage 0 sporulation protein YaaT	2.49E−02	−1.14	1			
2245148	OG1RF_12122	Stage 0 sporulation protein YaaT	3.40E−06	−1.65	1			
2245720	Intergenic_2182		4.03E−13	−1.51	1			
2345148	OG1RF_12225	Cold shock protein CspA	6.78E−53	−4.17	1			
2557127	OG1RF_12423	Trehalose operon repressor	2.38E−05	−1.03	1			
2567606	OG1RF_12434	DNA mismatch repair protein HexB	4.57E−04	−1.77	1	6.11E−15	−0.77	1
2571990	Intergenic_2504		3.36E−13	−1.10	1			
2682030	OG1RF_12531	CtsR family transcriptional regulator	1.97E−16	−3.00	1			
2682063	OG1RF_12531	CtsR family transcriptional regulator	3.75E−03	−1.88	1			
2738340	OG1RF_12576	Stage III sporulation protein J	2.80E−03	−1.02	1			

a BF/plank equals ratio of biofilm CFU/ml to planktonic CFU/ml.

We then sought to validate the importance of these genes for *in vitro* biofilm formation. However, large-scale testing of individual Tn mutants in CBRs is not feasible due to the volume of medium used for each reactor run (∼10 liters) as well as the physical size and processing time required for each sample set. Therefore, we chose three previously described *in vitro* experiments to validate biofilm phenotypes: (i) a 96-well plate assay in which biofilm biomass is stained and quantified relative to cell growth (23, 30), (ii) a submerged substrate assay in which biofilms are grown on an Aclar disc covered by growth medium (16, 26), and (iii) a miniature 96-well flow reactor system (MultiRep reactor) in which 96 samples can be cultured in a total of 12 channels on 5-mm disks (34). Because both M9 and TSB-D were used in the CBR TnSeq screen, we carried out the following experiments with both media.

### Phenotypes of “accessory” biofilm determinants in microtiter plate assays.

**FIG 2:**
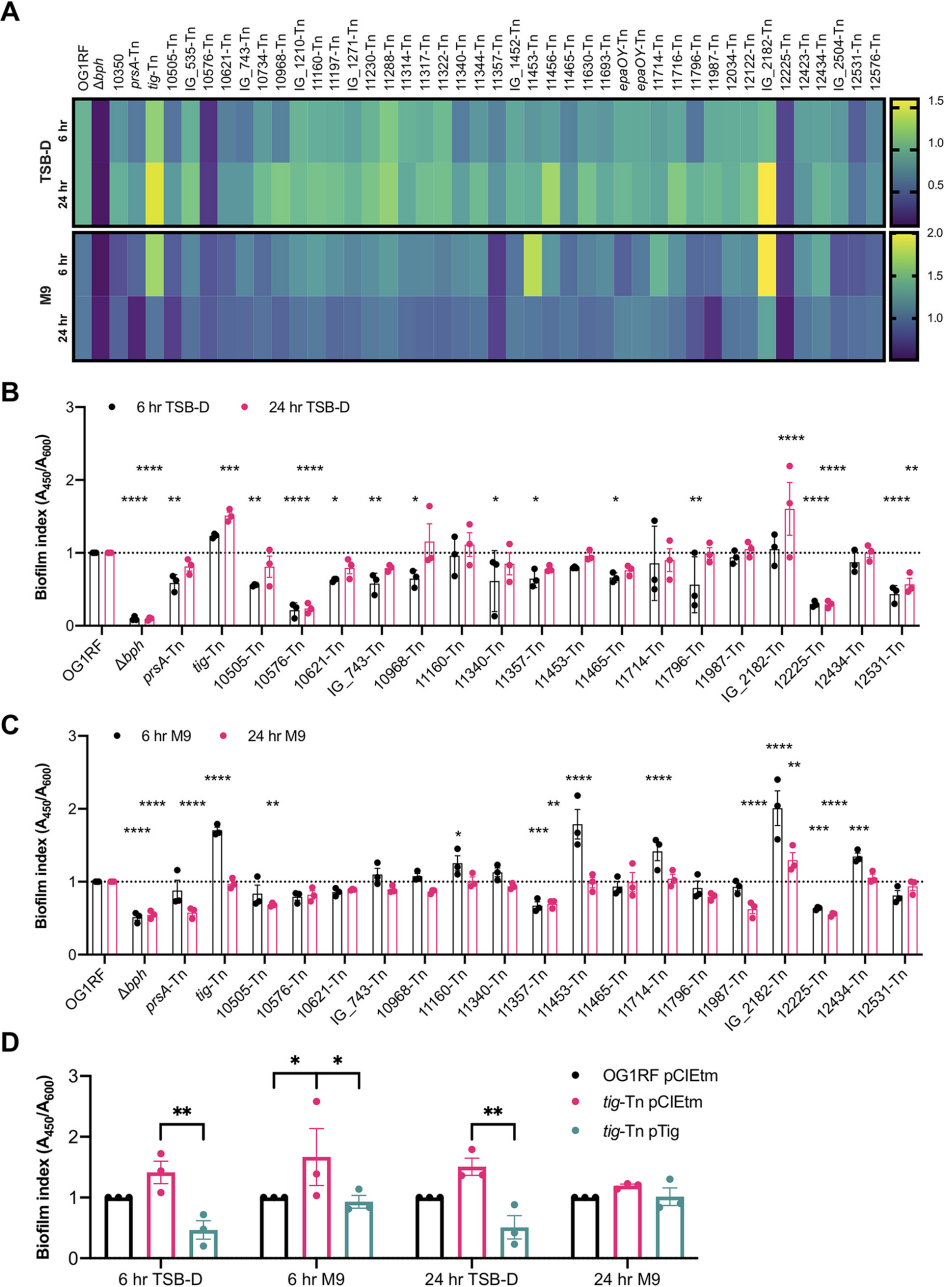
Tn mutants identified from biofilm TnSeq have variable biofilm production in microtiter plates. (A) Heat map summarizing biofilm index values (*A*_450_/*A*_600_ relative to OG1RF) for all mutants. Biofilm index shading legends are shown on the right. (B and C) TSB-D biofilm index values (B) and MM9-YEG biofilm index values (C) for all Tn mutants with significantly altered biofilm production in either medium. For clarity, a dotted line is shown at the OG1RF biofilm index value. Plotted values are the same ones represented in the heat maps in panel A. (D) Biofilm phenotypes were complemented for *tig*-Tn. Strains carried either an empty pCIEtm plasmid or pCIEtm with the wild-type allele cloned under a pheromone-inducible promoter. Biofilm assays were carried out in the growth medium and for the length of time indicated in *x*-axis labels. All cultures were grown with 25 ng/ml cCF10 to induce expression of the cloned *tig* gene. For panels B and C, three biological replicates were performed, each with two technical replicates. Statistical significance was evaluated by two-way analysis of variance (ANOVA) with Dunnett’s multiple-comparison test (*, *P* < 0.05; **, *P* < 0.01; ***, *P* < 0.001; ****, *P* < 0.0001). For panel D, three biological replicates were performed, each with three technical replicates. Statistical significance was evaluated by two-way ANOVA with Sidak’s multiple-comparison test (*, *P* < 0.05; **, *P* < 0.01; ***, *P* < 0.001; ****, *P* < 0.0001).

From the 55 Tn mutants presented in Table 3, we obtained 43 Tn mutants from the arrayed SmarT library stock plates. When multiple Tn insertions in a gene were identified, we chose only the insertion closest to the start codon. Additional mutations were excluded based on their location upstream of known biofilm determinants and the possibility that these insertions had polar effects on previously studied genes. To maintain consistency with previous experiments, we measured the biofilm production of the Tn mutants at 6 h and 24 h. A strain lacking *bph*, previously implicated in biofilm development (23), was used as a negative control, and biofilm production was normalized to that of OG1RF (Fig. 2A). In TSB-D, 12 mutants had significantly altered biofilm production relative to OG1RF at 6 h (12 decreased, 0 increased) (Fig. 2B, black bars), and 5 mutants had altered biofilm levels at 24 h (3 decreased, 2 increased) (Fig. 2B, pink bars). In MM9-YEG, 7 Tn mutants had altered biofilm production at 6 h (2 decreased, 5 increased) (Fig. 2C, black bars), and 6 mutants had altered biofilm levels at 24 h (5 decreased, 1 increased) (Fig. 2C, pink bars). Overall, ∼30% of mutants (13/43) had reduced biofilm formation relative to OG1RF. Interestingly, some mutant strains had higher biofilm production in MM9-YEG than TSB-D, including the Δ*bph* and OG1RF_10576 mutants, demonstrating that growth medium influences which genes are required for biofilm formation. We did not observe a correlation between the change in abundance (log_2_FC) of Tn mutants in TnSeq and the biofilm index in microtiter plate biofilm assays (Fig. S1E to H).

Although all 43 Tn mutants were underrepresented in biofilm TnSeq, ∼14% (6/43) had increased biofilm levels relative to that of OG1RF in 96-well plates (Fig. 2B and C). We chose to complement the high biofilm phenotype of the *tig*-Tn (OG1RF_10452-Tn) mutant by expression of the wild-type gene from a pheromone-inducible plasmid (23). *tig* encodes trigger factor, a chaperone involved in folding newly synthesized proteins (40). Expression of *tig* from a plasmid significantly decreased biofilm relative to the Tn mutant carrying an empty-vector plasmid (Fig. 2D). The opposing biofilm phenotypes observed for some Tn mutants in CBR TnSeq compared to 96-well plates underscores how determinants of biofilm formation may vary across experimental platforms and suggests that molecular changes during biofilm development are highly sensitive to specific assay conditions.

### Biofilm formation of Tn mutants in submerged substrate assays.

We chose 6 of the 43 Tn mutants described above for biofilm assays using submerged Aclar disc assays, in which strains are cultured in multiwell plates containing Aclar discs. These permit sampling of both planktonic and biofilm cells for visualization via microscopy and CFU quantification (16, 30). All 6 mutants were underrepresented in at least one library in biofilm TnSeq (Table 3; Tables S1 and S2) but had a range of phenotypes in the microtiter plate assays described above. Relative to parental OG1RF biofilm levels in 96-well plates, the *prsA*-Tn (encoding an extracellular peptidyl-prolyl isomerase [PPIase]) and OG1RF_10576-Tn (encoding a predicted DEAD box helicase) mutants had decreased biofilm. The *tig*-Tn (encoding trigger factor) mutant had increased biofilm, and OG1RF_10350-Tn, OG1RF_11456-Tn, and OG1RF_11288-Tn mutants did not have significantly different levels of biofilm compared to OG1RF (Fig. 2B and C).

**FIG 3:**
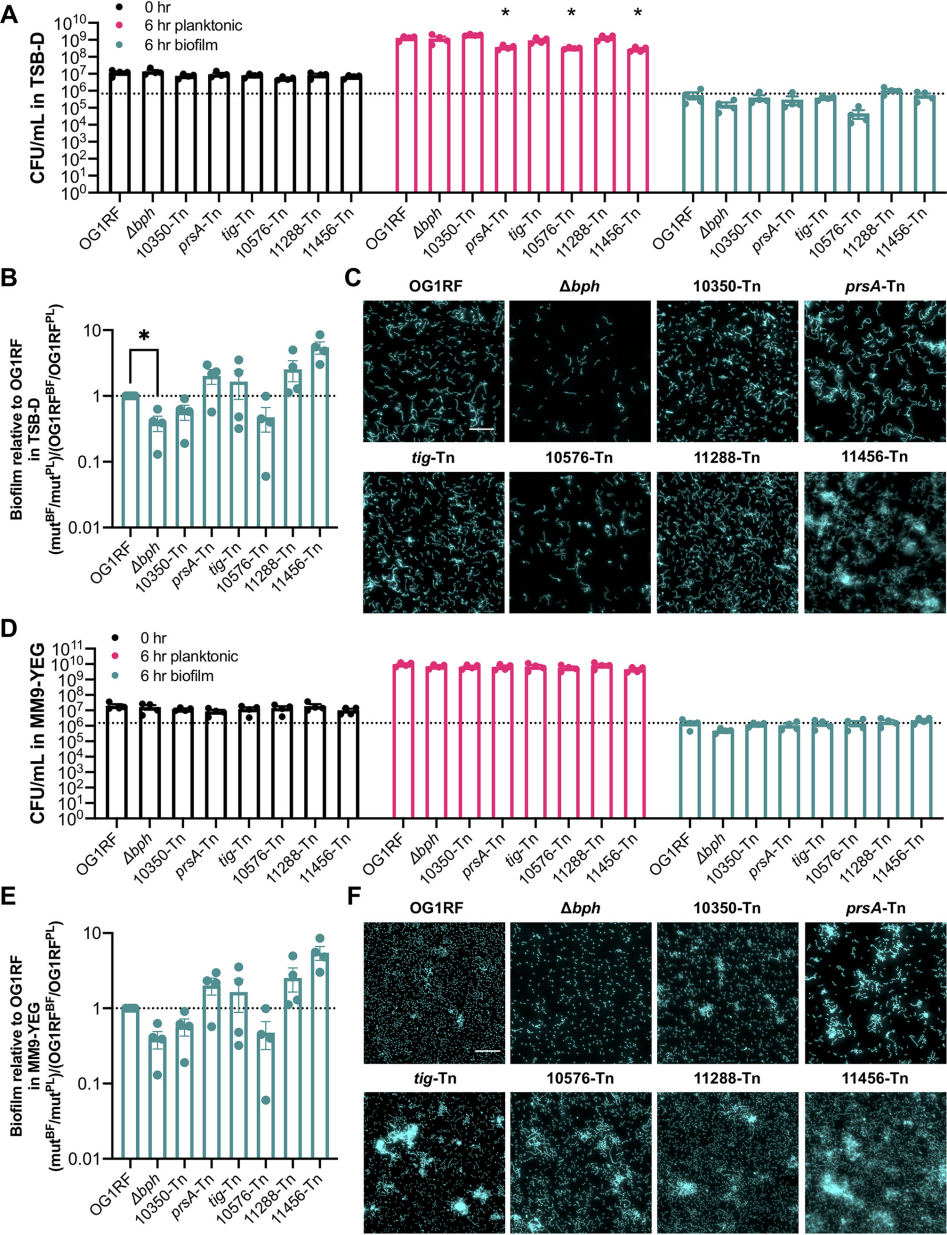
Biofilm formation of selected Tn mutants by use of a submerged Aclar disc assay. (A) Numbers of CFU/ml of strains at 0 h and 6 h in TSB-D. The dotted line indicates the number of OG1RF biofilm CFU/ml. (B) Ratio of biofilm (BF) growth to planktonic (PL) growth relative to that of OG1RF. (C) Representative microscopy images of Hoechst 33342-stained biofilms from TSB-D cultures. (D) Numbers of CFU/ml of strains at 0 h and 6 h in MM9-YEG. The dotted line indicates the number of OG1RF biofilm CFU/ml. (E) Ratio of biofilm growth to planktonic growth relative to that of OG1RF. (F) Representative microscopy images of Hoechst 33342-stained biofilms from MM9-YEG cultures. For panels A and D, each data point represents the average of two technical replicates, and a total of four biological replicates were performed. Statistical significance was evaluated by two-way ANOVA with Dunnett’s multiple-comparison test (*, *P* < 0.05; **, *P* < 0.01; ***, *P* < 0.001; ****, *P* < 0.0001). For panels B and E, values were obtained using the data points presented in panels A and D, respectively. Statistical significance was evaluated by one-way ANOVA with Dunnett’s multiple-comparison test (*, *P* < 0.05; **, *P* < 0.01; ***, *P* < 0.001; ****, *P* < 0.0001). For panels C and F, samples were grown in parallel to cultures used to generate panels A and D. Scale bars, 20 μm. Two technical replicates were processed for each biological replicate, and representative images are shown.

We inoculated strains at 10^7^ CFU/ml and quantified planktonic and biofilm CFU/ml after 6 h. In TSB-D, *prsA*-Tn, OG1RF_10576-Tn, and OG1RF_11456-Tn mutants had significantly lower numbers of planktonic CFU/ml than OG1RF (Fig. 3A, pink bars). The OG1RF_10576-Tn mutant had an ∼1 log decrease in biofilm CFU/ml relative to OG1RF (Fig. 3A, green bars), although this difference was not statistically significant. To determine whether mutants had a biofilm-specific decrease in viable cells (as opposed to lower biofilm growth due to growth defects in planktonic culture), we calculated the ratio of biofilm growth to planktonic growth relative to that of OG1RF. By this metric, only the Δ*bph* strain had a significant reduction relative to OG1RF (Fig. 3B).

Biofilms were visualized with fluorescence microscopy after staining with Hoechst 33342, a nucleic acid label. OG1RF biofilms consistently grew as a monolayer of short chains of bacteria with few multicellular aggregates or clumps (Fig. 3C). As previously observed, biofilms formed by the Δ*bph* negative-control strain contained fewer cells than OG1RF (23). The appearance of OG1RF_10350-Tn, *tig*-Tn, and OG1RF_11288-Tn biofilms was similar to that of OG1RF. Although there was not a significant reduction in OG1RF_10576-Tn biofilm CFU relative to OG1RF (Fig. 3B), these mutant biofilms had visibly less surface coverage than OG1RF biofilms. *prsA*-Tn biofilms contained some multicellular aggregates, and OG1RF_11456-Tn biofilms had large clumps of cells (Fig. 3C).

We next examined the growth of these mutants in MM9-YEG. Unlike the corresponding experiments in TSB-D (Fig. 3A, pink bars), no mutants had reduced numbers of CFU/ml in planktonic culture (Fig. 3D, pink bars). Additionally, none of the mutants had reduced numbers of CFU/ml in biofilms (Fig. 3D, green bars) or ratio of biofilm growth to planktonic growth relative to OG1RF (Fig. 3E). However, visualization of Aclar substrates revealed substantial differences in biofilm architecture. In MM9-YEG, OG1RF formed a monolayer biofilm composed mainly of single cells and some small aggregates (Fig. 3F). The Δ*bph* biofilm had less surface coverage but was still composed of mostly single cells. All Tn mutants formed biofilms with multicellular aggregates. *prsA*-Tn, *tig*-Tn, and OG1RF_10576-Tn biofilms had mixtures of single cells and small multicellular chains, while nearly all cells in OG1RF_11456-Tn biofilms grew as chains and aggregates. Interestingly, fewer multicellular chains and more individual cells were observed in biofilms grown in MM9-YEG than in TSB-D (compare Fig. 3C and F). Conversely, more large multicellular aggregates were observed in MM9-YEG than in TSB-D, suggesting that nutritional components could regulate cell chaining and aggregate formation as separate processes during biofilm growth.

### Biofilm formation in miniature flow reactors.

**FIG 4:**
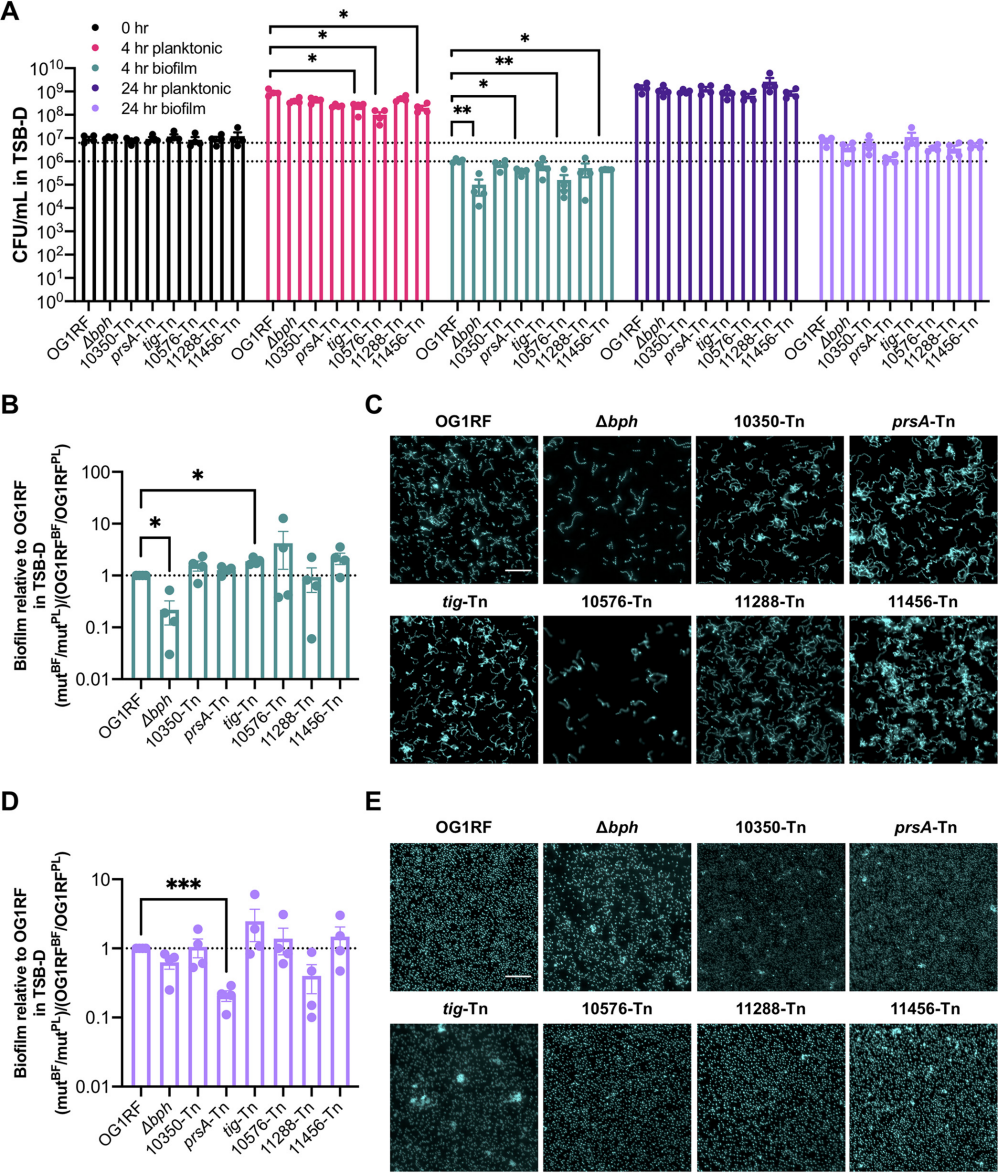
Biofilm formation of selected Tn mutants grown in MultiRep reactors in TSB-D. (A) Numbers of CFU/ml of strains at 0 h, 4 h, and 24 h. The dotted lines indicate the numbers of OG1RF biofilm CFU/ml at 24 h (top line) and 4 h (bottom line). (B) Ratio of biofilm growth to planktonic growth at 4 h relative to that of OG1RF. (C) Representative microscopy images of Hoechst 33342-stained biofilms at 4 h. (D) Ratio of biofilm growth to planktonic growth at 24 h relative to that of OG1RF. (E) Representative microscopy images of Hoechst 33342-stained biofilms at 24 h. For panel A, each data point represents the average of two technical replicates, and a total of four biological replicates were performed. For panels B and D, data points were derived using the data points shown in panel A. Statistical significance was evaluated by one-way ANOVA with Dunnett’s multiple-comparison test (*, *P*  < 0.05; **, *P*  < 0.01; ***, *P*  < 0.001; ****, *P*  < 0.0001). For panels C and E, samples were grown in parallel to cultures used to generate panel A. Scale bars, 20 μm. Two technical replicates were processed for each biological replicate, and representative images are shown.

MultiRep reactors are miniaturized 12-channel biofilm flow reactors that permit simultaneous sampling of planktonic cultures and biofilms formed on removable Aclar discs that rest in wells in each channel (Fig. S2A). OG1RF biofilms from MultiRep reactors resemble the monolayer biofilms formed in CBRs (14, 16, 34). The same 6 Tn mutant cultures used for submerged Aclar disc assays in the previous section were inoculated into the MultiRep reactors at 10^7^ CFU/ml and grown with static incubation for 4 h, after which medium flowed through each channel at a rate of 0.1 ml/min for 20 h (*t*_total_ = 24 h). The flow rate for growth medium was chosen for consistency in turnover rate compared to CBR experiments. Planktonic and biofilm cultures were quantified and visualized at 4 h and 24 h. After 4 h of growth in TSB-D, planktonic cultures of *tig*-Tn, OG1RF_10576-Tn, and OG1RF_11456-Tn strains had significantly reduced numbers of CFU/ml relative to OG1RF (Fig. 4A, pink bars). The Δ*bph* negative-control strain had significantly reduced numbers of biofilm CFU/ml relative to OG1RF, as did the *prsA*-Tn, OG1RF_10576-Tn, and OG1RF_11456-Tn mutants (Fig. 4A, green bars). However, only the Δ*bph* strain had a biofilm-specific reduction in growth relative to OG1RF at 4 h (Fig. 4B). The *tig*-Tn mutant, which had increased biofilm formation in microtiter plate assays (Fig. 2D), had a biofilm-specific 1.89-fold ml relative to OG1RF/ml relative to OG1RF (Fig. 4B).

10.1128/mBio.01011-21.2 FIG S2 MultiRep biofilm reactors and analysis of OG1RF biofilms grown under multiple experimental conditions. (A) Photograph showing an assembled MultiRep biofilm reactor. Bottles with sterile growth medium are shown on the left, and outflow tubes with waste containers are shown on the right. (B) Additional fluorescence microscopy images of OG1RF biofilms obtained during biological replicates of experiments shown in Fig. 3, 4, and 5. Scale bars, 20 μm. (C to E) Images of OG1RF biofilms were used for Comstat2 analysis of overall biomass (C), average biofilm thickness (D), and maximum biofilm thickness (E). Statistical significance was evaluated by two-way ANOVA with Tukey’s multiple-comparison test (*, *P* < 0.05; **, *P* < 0.01; ***, *P* < 0.001; ****, *P* < 0.0001) Download FIG S2, TIF file, 1.5 MB. Copyright © 2021 Willett et al. 2021 Willett et al. https://creativecommons.org/licenses/by/4.0/ This content is distributed under the terms of the Creative Commons Attribution 4.0 International license.

Biofilm appearance was evaluated using fluorescence microscopy of Hoescht 33342-stained cells. After 4 h, OG1RF formed biofilms with single cells and multicell chains but few large aggregates (Fig. 4C). Biofilms formed by Δ*bph* and OG1RF_10576-Tn strains had very few cells, in agreement with the average reduction in biofilm CFU/ml at 4 h. OG1RF_10350-Tn and *tig*-Tn mutants formed biofilms with chained cells and small clumps, and *prsA*-Tn and OG1RF_11456-Tn mutants formed biofilms with larger clumps of cells. The OG1RF_11288-Tn mutant formed biofilms that resembled those of OG1RF.

After 24 h, no mutants had reduced numbers of planktonic CFU/ml relative to OG1RF (Fig. 4A, dark purple bars). Although the number of biofilm CFU/ml of the *prsA*-Tn mutant was ∼1 log lower than that of OG1RF (Fig. 4A, lilac bars), this difference was not statistically significant. However, the *prsA*-Tn mutant had a significant reduction in the ratio of biofilm cells to planktonic cells relative to OG1RF (Fig. 4D). In contrast to biofilm morphology at 4 h, OG1RF biofilms at 24 h appeared as smooth layers of single cells, and chaining and clumping were not evident (Fig. 4E; Fig. S2B). Unlike 4-h biofilms formed by Δ*bph* and OG1RF_10576-Tn strains, biofilms after 24 h of growth covered most of the Aclar surface. OG1RF_10350-Tn and OG1RF_11288-Tn biofilms resembled those of OG1RF, and small clumps of cells were visible in *prsA*-Tn, *tig*-Tn, and OG1RF_11456-Tn biofilms.

**FIG 5:**
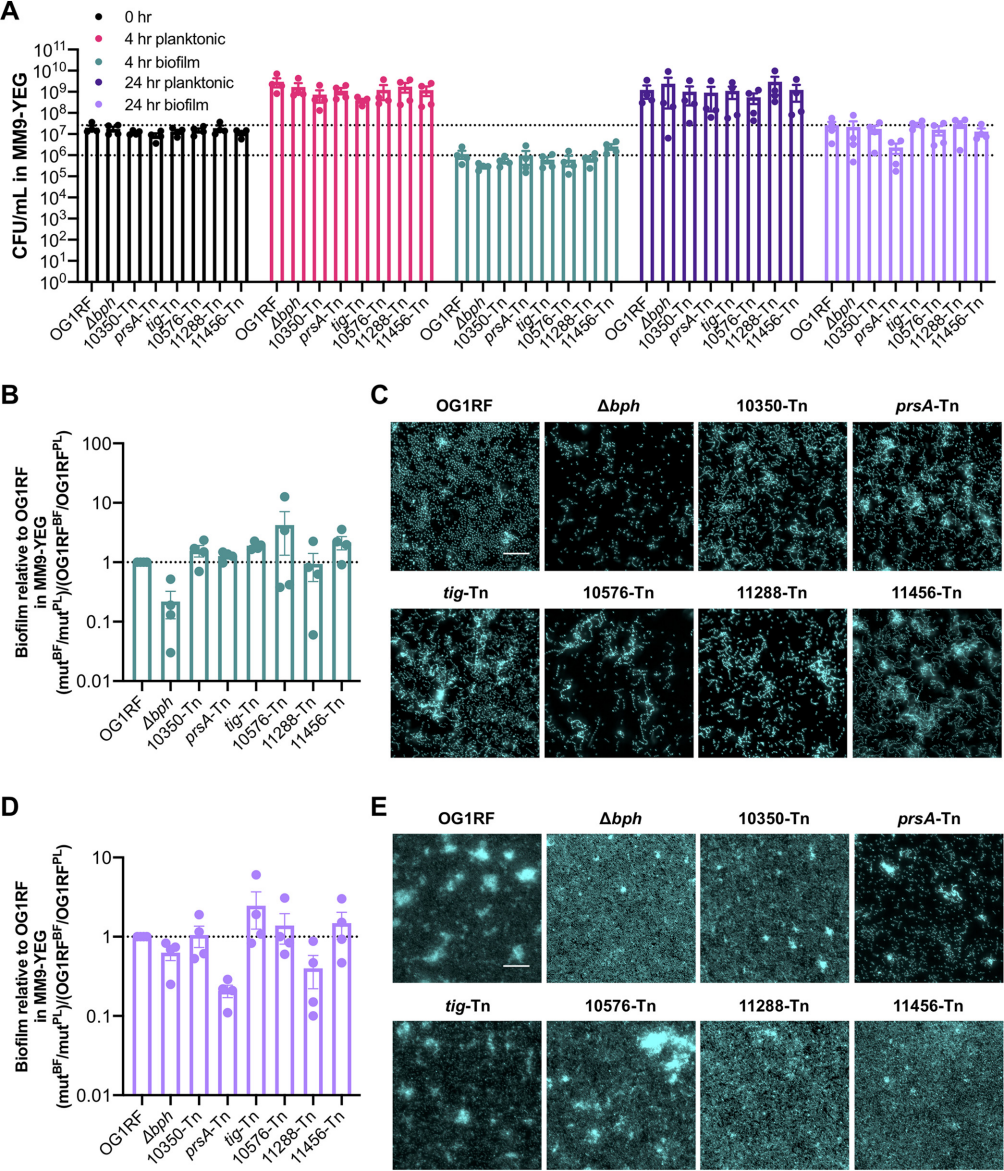
Biofilm formation of selected Tn mutants grown in MultiRep reactors in MM9-YEG. (A) Numbers of CFU/ml of strains at 0 h, 4 h, and 24 h. The dotted lines indicate numbers of OG1RF biofilm CFU/ml at 24 h (top line) and 4 h (bottom line). (B) Ratio of biofilm growth to planktonic growth at 4 h relative to that of OG1RF. (C) Representative microscopy images of Hoechst 33342-stained biofilms at 4 h. (D) Ratio of biofilm growth to planktonic growth at 24 h relative to that of OG1RF. (E) Representative microscopy images of Hoechst 33342-stained biofilms at 24 h. For panel A, each data point represents the average of two technical replicates, and a total of four biological replicates were performed. For panels B and D, data points were derived using the data points shown in panel A. Statistical significance was evaluated by one-way ANOVA with Dunnett’s multiple-comparison test. For panels C and E, samples were grown in parallel to cultures used to generate panel A. Scale bars, 20 μm. Two technical replicates were processed for each biological replicate, and representative images are shown.

In MM9-YEG, no mutants had statistically different numbers of planktonic or biofilm CFU/ml compared to those of OG1RF after 4 h of static growth (Fig. 5A, pink and green bars) or an additional 20 h of growth under flow conditions (Fig. 5A, purple and lilac bars). We observed more variability in planktonic growth of each Tn mutant after 24 h in MM9-YEG than in TSB-D. Accordingly, no strains had biofilm-specific decreases in CFU calculated as the ratio of biofilm growth to planktonic growth relative to OG1RF (Fig. 5B and D). Despite the variability in CFU, morphological differences in biofilms were visible. After 4 h, OG1RF biofilms grew as single cells with small clumps (Fig. 5C). OG1RF_10350-Tn biofilms had fewer individual cells and more small chains than OG1RF. Reduced surface coverage was observed in Δ*bph* and OG1RF_10576-Tn biofilms, and OG1RF_10576-Tn biofilms had long chains of cells relative to OG1RF. *prsA*-Tn, *tig*-Tn, and OG1RF_11456-Tn biofilms all had large aggregates of cells. After 24 h, OG1RF formed dense, thick biofilms with visible cellular aggregates (Fig. 5E). Biofilms formed by Δ*bph*, OG1RF_10350-Tn, OG1RF_11456-Tn, OG1RF_11288-Tn strains had some small aggregates. *prsA*-Tn and *tig*-Tn biofilms had sparse surface coverage with large clusters of cells, and OG1RF_10576-Tn formed biofilms with large aggregates.

### Comparative measurements of biofilm growth of OG1RF in different growth assays.

Because we observed differences in biofilm morphology depending on growth medium, we used Comstat2 (41) to quantify the biomass and thickness of the parental strain by using submerged Aclar disc (6-h) and MultiRep reactor (4-h and 24-h) assays. In general, biofilms grown in MM9-YEG contained more individual cells, whereas biofilms grown in TSB-D had more multicellular chains (Fig. 3C and F, 4C and E, and 5C and E; Fig. S2B). In TSB-D, biomass was not significantly different between submerged Aclar and MultiRep biofilms, and biomass of submerged Aclar or 4-h MultiRep biofilms grown in TSB-D was not significantly different from those grown in MM9-YEG (Fig. S2C). However, the biomass of 24-h MultiRep biofilms grown in MM9-YEG was 5.3-fold greater than that of biofilms grown in TSB-D (Fig. S2C). In MM9-YEG, 24-h MultiRep biofilms also had more biomass than 6-h submerged Aclar biofilms (3.45-fold higher) and 4-h MultiRep biofilms (13.0-fold higher) (Fig. S2C).

We next measured biofilm thickness. Biofilms grown on submerged Aclar discs for 6 h or the MultiRep reactor for 4 h had similar average thicknesses regardless of growth medium (Fig. S2D). However, biofilms grown in the MultiRep reactor for 24 h in MM9-YEG had an average thickness of 23.3 μm, which is 4.06-fold higher than the average thickness of biofilms grown in TSB-D (5.74 μm) and also significantly higher than the other biofilms grown in MM9-YEG (Fig. S2D). All biofilms grown in TSB-D had approximately the same maximum thickness (Fig. S2E). However, 24-h MultiRep biofilms grown in MM9-YEG had a maximum thickness of 27.7 μm, which is ∼2-fold more than the other MM9-YEG biofilms and ∼2.5-fold greater than biofilms grown in TSB-D. Taken together, these measurements show that extended cultivation of OG1RF biofilms in MM9-YEG under flow conditions results in thicker biofilms with more biomass than that in TSB-D, which correlates with the qualitative assessment of biofilm morphology observed using fluorescence microscopy. However, it is currently unknown whether this increase is due solely to the presence of more biofilm cells or to changes in matrix production or composition.

### Tn mutant competition against OG1RF in biofilm cocultures.

**FIG 6:**
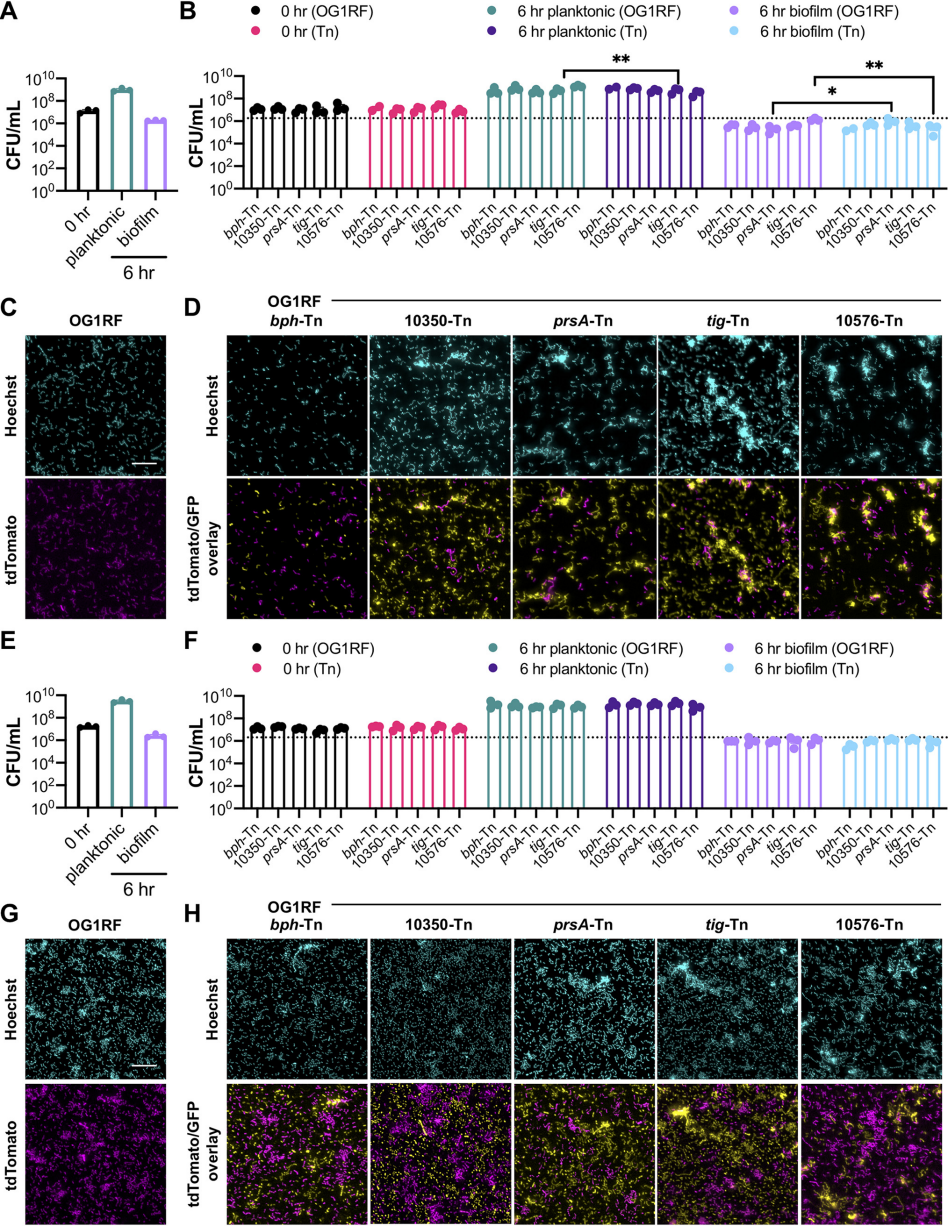
Cocultures of OG1RF and Tn mutants using the submerged Aclar disc assay. (A) Numbers of CFU/ml of OG1RF grown in TSB-D at 0 h and 6 h. (B) Numbers of CFU/ml of OG1RF/Tn cocultures grown in TSB-D at 0 h and 6 h. The dotted line indicates the number of biofilm CFU/ml of OG1RF grown in monoculture (value taken from panel A). (C) Representative microscopy images of Hoechst 33342-stained OG1RF pP_23_::tdTomato biofilms grown in TSB-D at 6 h. (D) Representative microscopy images of Hoechst 33342-stained OG1RF pP_23_::tdTomato/Tn mutant pP_23_::GFP biofilms grown in TSB-D at 6 h. (E) Numbers of CFU/ml of OG1RF grown in MM9-YEG at 0 h and 6 h. (F) Numbers of CFU/ml of OG1RF/Tn cocultures grown in MM9-YEG at 0 h and 6 h. The dotted line indicates the number of biofilm CFU/ml of OG1RF grown in monoculture (value taken from panel E). (G) Representative microscopy images of Hoechst 33342-stained OG1RF pP_23_::tdTomato biofilms grown in MM9-YEG at 6 h. (H) Representative microscopy images of Hoechst 33342-stained OG1RF pP_23_::tdTomato/Tn mutant pP_23_::GFP biofilms grown in MM9-YEG at 6 h. For panels A, B, E, and F, each data point represents the average of two technical replicates, and a total of four biological replicates were performed. Statistical significance was evaluated by two-way ANOVA with Sidak’s multiple-comparison test (*, *P* < 0.05; **, *P* < 0.01; ***, *P* < 0.001; ****, *P* < 0.0001). For panels C, D, G, and H, samples were grown in parallel to cultures used to generate panels A, B, E, and F. Scale bars, 20 μm. Two technical replicates were processed for each biological replicate, and representative images are shown.

**FIG 7:**
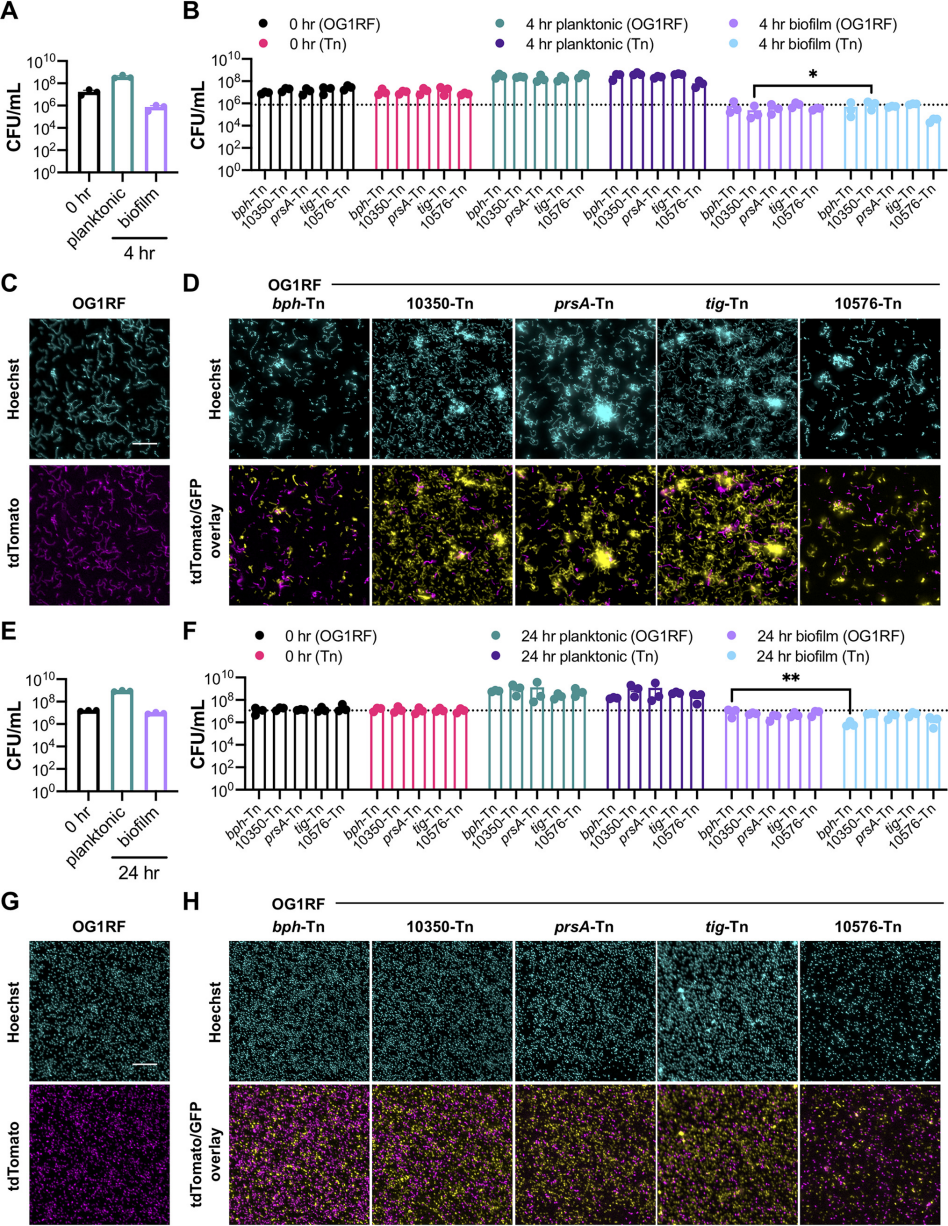
Cocultures of OG1RF and Tn mutants in TSB-D in MultiRep reactors. (A) Numbers of CFU/ml of OG1RF at 0 h and 4 h. (B) Numbers of CFU/ml of OG1RF/Tn cocultures at 0 h and 4 h. The dotted line indicates the number of biofilm CFU/ml of OG1RF grown in monoculture (value taken from panel A). (C) Representative microscopy images of Hoechst 33342-stained OG1RF pP_23_::tdTomato biofilms at 4 h. (D) Representative microscopy images of Hoechst 33342-stained OG1RF pP_23_::tdTomato/Tn mutant pP_23_::GFP biofilms at 4 h. (E) Numbers of CFU/ml of OG1RF at 0 h and 24 h. (F) Numbers of CFU/ml of OG1RF/Tn cocultures at 0 h and 24 h. The dotted line indicates the number of biofilm CFU/ml of OG1RF grown in monoculture (value taken from panel E). (G) Representative microscopy images of Hoechst 33342-stained OG1RF pP_23_::tdTomato biofilms at 24 h. (H) Representative microscopy images of Hoechst 33342-stained OG1RF pP_23_::tdTomato/Tn mutant pP_23_::GFP biofilms at 24 h. For panels A, B, E, and F, each data point represents the average of two technical replicates, and a total of three biological replicates were performed. Statistical significance was evaluated by two-way ANOVA with Sidak’s multiple-comparison test (*, *P* < 0.05; **, *P*  < 0.01; ***, *P* < 0.001; ****, *P*  < 0.0001). For panels C, D, G, and H, samples were grown in parallel to cultures used to generate panels A, B, E, and F. Scale bars, 20 μm. Two technical replicates were processed for each biological replicate, and representative images are shown.

**FIG 8:**
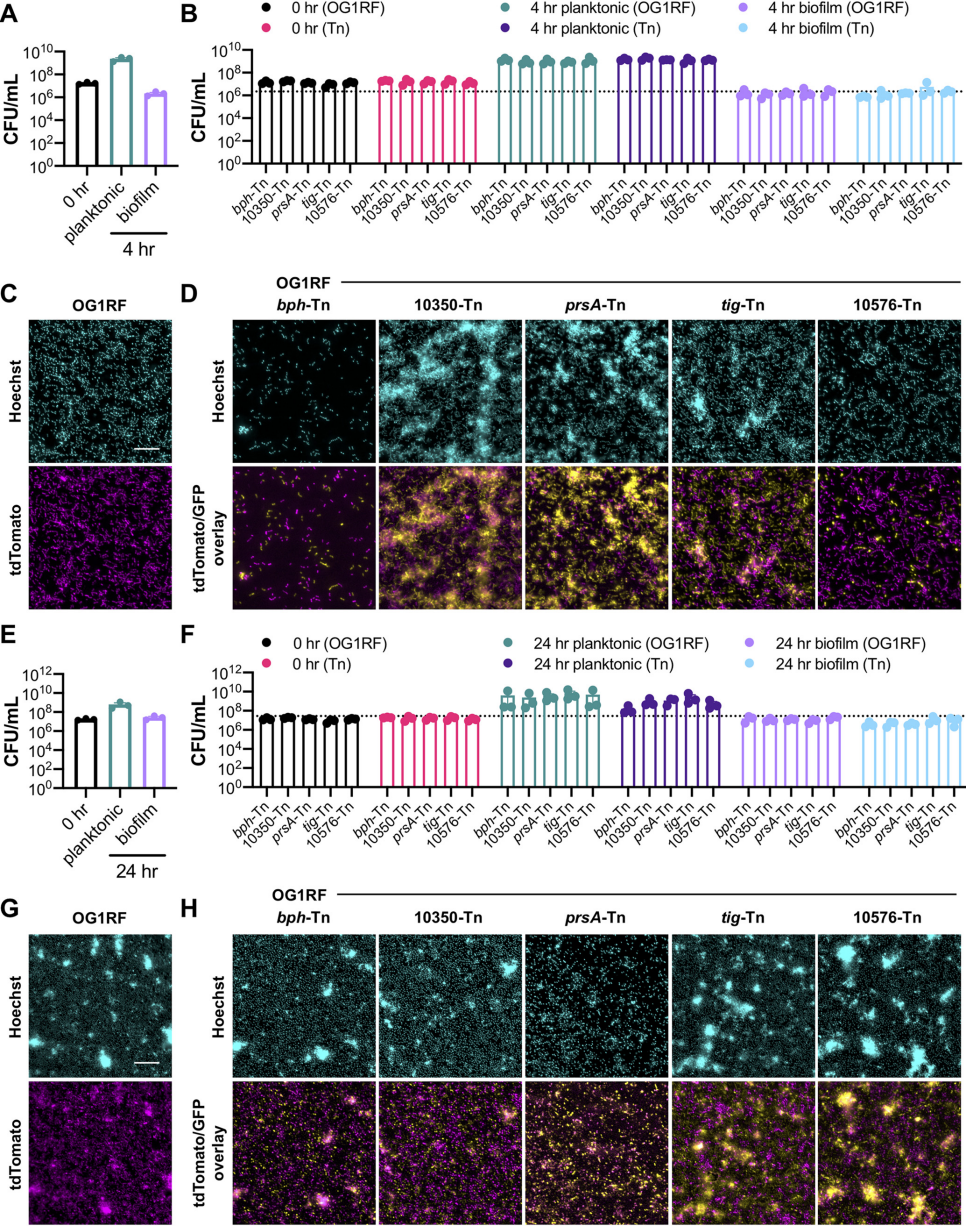
Cocultures of OG1RF and Tn mutants in MM9-YEG in MultiRep reactors. (A) Numbers of CFU/ml of OG1RF at 0 h and 4 h. (B) Numbers of CFU/ml of OG1RF/Tn cocultures at 0 h and 4 h. The dotted line indicates the number of biofilm CFU/ml of OG1RF grown in monoculture (value taken from panel A). (C) Representative microscopy images of Hoechst 33342-stained OG1RF pP_23_::tdTomato biofilms at 4 h. (D) Representative microscopy images of Hoechst 33342-stained OG1RF pP_23_::tdTomato/Tn mutant pP_23_::GFP biofilms at 4 h. (E) Numbers of CFU/ml of OG1RF at 0 h and 24 h. (F) Numbers of CFU/ml of OG1RF/Tn cocultures at 0 h and 24 h. The dotted line indicates the number of biofilm CFU/ml of OG1RF grown in monoculture (value taken from panel E). (G) Representative microscopy images of Hoechst 33342-stained OG1RF pP_23_::tdTomato biofilms at 24 h. (H) Representative microscopy images of Hoechst 33342-stained OG1RF pP_23_::tdTomato/Tn mutant pP_23_::GFP biofilms at 24 h. For panels A, B, E, and F, each data point represents the average of two technical replicates, and a total of three biological replicates were performed. Statistical significance was evaluated by two-way ANOVA with Sidak’s multiple-comparison test (*, *P*  < 0.05; **, *P*  < 0.01; ***, *P*  < 0.001; ****, *P*  < 0.0001). For panels C, D, G, and H, samples were grown in parallel to cultures used to generate panels A, B, E, and F. Scale bars, 20 μm. Two technical replicates were processed for each biological replicate, and representative images are shown.

The 6 Tn mutants described above were originally identified using TnSeq to evaluate mutant abundance in a community. Therefore, we wanted to measure how the mutants competed in a coculture with parental OG1RF. In the data reported below, we used both enumeration on selective agar medium (Tn mutants are resistant to chloramphenicol) and fluorescence microscopy to analyze the results of cocultures. For enumeration, we replaced the Δ*bph* negative control with the *bph*-Tn mutant, which has the same biofilm phenotype as the deletion strain (23). To differentially label strains for visualization, we transformed OG1RF with a plasmid expressing tdTomato from a strong constitutive promoter (pP_23_::tdTomato) and each Tn mutant with a plasmid expressing P_23_::GFP. Prior to coculture, we evaluated whether carriage of the tdTomato or green fluorescent protein (GFP) plasmids resulted in growth defects. Two mutants (OG1RF_11456-Tn and OG1RF_11288-Tn) were excluded from coculture experiments due to poor planktonic growth or unstable fluorescence. With the remaining 4 Tn mutants, we repeated the submerged Aclar disc experiments described above with cultures in which OG1RF was mixed with single Tn mutants. For all experiments, OG1RF pP_23_::tdTomato was also cultured independently in addition to coculture with Tn mutants to ensure that expression of tdTomato did not negatively affect biofilm formation (Fig. 6A, C, E, and G; Fig. 7A, C, E, and G; Fig. 8A, C, E, and G).

For submerged Aclar disc assays, we inoculated both strains at 10^7^ CFU/ml and quantified OG1RF and Tn mutants after 6 h. In planktonic cultures grown in TSB-D, only the OG1RF-10576-Tn mutant had a significant difference in number of CFU/ml (∼1 log decrease) relative to OG1RF in the same coculture (Fig. 6B). The number of biofilm CFU/ml of OG1RF_10576-Tn was also decreased to the same extent relative to OG1RF in coculture. Interestingly, the *prsA*-Tn mutant outgrew OG1RF in these coculture biofilms by ∼1 log (Fig. 6B) and had a 4.23-fold increase in the ratio of biofilm CFU to planktonic CFU relative to OG1RF (Fig. S3C), suggesting that this mutant outcompeted OG1RF under these conditions. Cocultures were visualized by fluorescence microscopy (Fig. 6C and D; Fig. S3A). *bph*-Tn/OG1RF biofilms had sparse surface coverage compared to that of OG1RF alone. The OG1RF_10350-Tn/OG1RF biofilm resembled biofilms formed by the individual strains grown in monoculture. In accordance with CFU quantification, the *prsA*-Tn/OG1RF biofilm had more *prsA*-Tn cells and small clumps than that of OG1RF. In contrast to *tig*-Tn monoculture biofilms, the *tig*-Tn mutant formed large clumps in coculture with OG1RF. OG1RF_10576-Tn biofilms had low surface coverage when cultured alone, yet this mutant formed large clumps when cocultured with OG1RF. Interestingly, these large clusters appeared to colocalize with patches of OG1RF cells (Fig. S3A).

10.1128/mBio.01011-21.3 FIG S3 Individual channels and relative biofilm growth of Tn mutants in submerged Aclar disc cocultures. (A and B) The individual tdTomato and GFP panels for TSB-D cocultures (A) and MM9-YEG cocultures (B) that are shown as overlays in Fig. 6D and H are presented here for clarity. Scale bars, 20 μm. (C and D) The ratios of biofilm growth to planktonic growth relative to that of OG1RF were calculated for TSB-D cocultures (C) and MM9-YEG cocultures (D). Data points in panels C and D were calculated from the CFU/ml values presented in Fig. 6. Statistical significance was evaluated by two-way ANOVA with Dunnett’s multiple-comparison test (*, *P* < 0.05; **, *P* < 0.01; ***, *P* < 0.001; ****, *P* < 0.0001). Download FIG S3, TIF file, 1.7 MB. Copyright © 2021 Willett et al. 2021 Willett et al. https://creativecommons.org/licenses/by/4.0/ This content is distributed under the terms of the Creative Commons Attribution 4.0 International license.

In MM9-YEG, none of the mutants had significantly different numbers of planktonic or biofilm CFU/ml than OG1RF (Fig. 6F). Overall, the MM9-YEG biofilms had more surface coverage than the TSB-D biofilms (compare Fig. 6C and D and Fig. 6G and H), and all strains had higher numbers of biofilm CFU/ml in MM9-YEG than in TSB-D. The *bph*-Tn/OG1RF and OG1RF_10350-Tn/OG1RF biofilms resembled those of the mutants and OG1RF grown individually (Fig. 6G and H; Fig. S3B). However, *prsA*-Tn, *tig*-Tn, and OG1RF_10576-Tn biofilms contained fewer aggregates when cocultured with OG1RF than when grown individually. Additionally, biofilms from the *tig*-Tn mutant cocultured with OG1RF in MM9-YEG contained more individual *tig*-Tn cells (as opposed to multicellular chains) than when cocultured in TSB-D. The OG1RF_10576-Tn mutant formed clumps and chains with visibly less surface coverage than OG1RF (Fig. S3B) when cocultured with OG1RF in MM9-YEG, although there was no statistical difference between numbers of OG1RF_10576-Tn and OG1RF biofilm CFU/ml.

### Biofilm formation of OG1RF and Tn mutant cocultures in miniature flow reactors.

Biofilm formation of cocultures was evaluated using the MultiRep biofilm flow chambers described above, and each strain was inoculated at 10^7^ CFU/ml. After 4 h in TSB-D, there were no statistically significant differences in numbers of planktonic CFU/ml between OG1RF and any mutants, but OG1RF_10350-Tn/OG1RF biofilms contained 3.6-fold more OG1RF_10350-Tn CFU than OG1RF CFU (Fig. 7B). Visualization of biofilms revealed that OG1RF_10350 and *tig*-Tn mutants formed biofilms with aggregates containing both mutant and OG1RF cells (Fig. 7D; Fig. S4A). The *prsA*-Tn mutant formed large aggregates in coculture with OG1RF, but these aggregates contained relatively few OG1RF cells (Fig. 7D; Fig. S4A). *bph*-Tn/OG1RF and OG1RF_10576-Tn/OG1RF biofilms had less surface coverage than OG1RF grown alone (Fig. 7C and D). After 24 h of growth in TSB-D, there were no significant differences in numbers of coculture planktonic or biofilm CFU/ml (Fig. 7F). OG1RF pP_23_::tdTomato and coculture biofilms grew as monolayers of mostly individual cells, with fewer multicellular aggregates and less chaining than observed after 4 h (Fig. 7G and H). Fewer *bph*-Tn and OG1RF_10576-Tn cells were present than OG1RF cells (Fig. 7H; Fig. S4B), although only the *bph*-Tn mutant had significantly reduced numbers of biofilm CFU/ml relative to OG1RF (Fig. 7F).

10.1128/mBio.01011-21.4 FIG S4 Individual channels and relative biofilm growth of Tn mutants in TSB-D in MultiRep reactors. (A and B) The individual tdTomato and GFP panels for 4-h (A) and 24-h (B) cocultures that are shown as overlays in Fig. 7D and H are presented here for clarity. Scale bars, 20 μm. (C and D) The ratios of biofilm growth to planktonic growth relative to that of OG1RF were calculated for 4-h (C) and 24-h (D) cocultures. Data points in panels C and D were calculated from the CFU/ml values presented in Fig. 7. Statistical significance was evaluated by two-way ANOVA with Dunnett’s multiple-comparison test (*, *P* < 0.05; **, *P* < 0.01; ***, *P* < 0.001; ****, *P* < 0.0001). Download FIG S4, TIF file, 2.2 MB. Copyright © 2021 Willett et al. 2021 Willett et al. https://creativecommons.org/licenses/by/4.0/ This content is distributed under the terms of the Creative Commons Attribution 4.0 International license.

After 4 h in MM9-YEG, there were no significant differences in numbers of planktonic or biofilm CFU/ml between OG1RF and Tn mutants in coculture (Fig. 8B). Very few mutant cells were visible in the *bph*-Tn/OG1RF and OG1RF_10576-Tn/OG1RF biofilms (Fig. 8D; Fig. S5A). OG1RF_10350/OG1RF biofilms had larger aggregates of cells than those grown in TSB-D for 4 h. *prsA*-Tn/OG1RF and *tig*-Tn/OG1RF biofilms resembled those grown in TSB-D for 4 h and contained large aggregates of cells. After 24 h of growth in MM9-YEG, there were no significant differences between numbers of OG1RF or Tn mutant CFU/ml in planktonic or biofilm cultures (Fig. 8F). Coculture biofilms contained thick multicellular aggregates of both OG1RF and Tn mutants, with the exception of *prsA*-Tn coculture biofilms, which had fewer large aggregates (Fig. 8H; Fig. S5B). None of the Tn mutants had significant differences in the ratio of biofilm cells to planktonic cells relative to OG1RF at either 4 h or 24 h (Fig. S5C and D).

10.1128/mBio.01011-21.5 FIG S5 Individual channels and relative biofilm growth of Tn mutants in MM9-YEG in MultiRep reactors. (A and B) The individual tdTomato and GFP panels for 4-h (A) and 24-h (B) cocultures that are shown as overlays in Fig. 8D and H are presented here for clarity. Scale bars, 20 μm. (C and D) The ratios of biofilm growth to planktonic growth relative to that of OG1RF were calculated for 4-h (C) and 24-h (D) cocultures. Data points in panels C and D were calculated from the CFU/ml values presented in Fig. 8. Statistical significance was evaluated by two-way ANOVA with Dunnett’s multiple-comparison test (*, *P* < 0.05; **, *P* < 0.01; ***, *P* < 0.001; ****P < 0.0001). Download FIG S5, TIF file, 2.3 MB. Copyright © 2021 Willett et al. 2021 Willett et al. https://creativecommons.org/licenses/by/4.0/ This content is distributed under the terms of the Creative Commons Attribution 4.0 International license.

### Putative biochemical activities of newly identified biofilm determinants from structural modeling and a functional assay.

Between 10 and 40% of bacterial gene products are poorly characterized or annotated as hypothetical (42), although they are frequently identified as loci of interest in experiments in E. faecalis OG1RF and other organisms (22, 30, 43, 44). Of the 45 new genes identified as biofilm determinants from TnSeq (Table 3), 6 were annotated as hypothetical, as encoding gene products that are incongruous with known E. faecalis biology (chemotaxis or sporulation), or had conflicting annotations across multiple databases (NCBI and KEGG). Others had vague annotations, and their function had not been studied in *Enterococcus*. We used Phyre2 (45) to predict structures for 14 proteins for which we tested the corresponding Tn mutants in 96-well plate biofilm assays (Table S3), including 3 chosen for analysis with microscopy and cocultures (OG1RF_10350-Tn, OG1RF_11288-Tn, and OG1RF_11456). OG1RF_10350 and OG1RF_11288 are annotated in different databases as LytR-Cps2a-Psr (LCP) family proteins or transcriptional regulators. Early studies on LCP family proteins suggested that they could be transcription factors, but the well-characterized examples are phosphotransferases that catalyze attachment of glycopolymers to the cell wall of Gram-positive bacteria (46). OG1RF_10350 and OG1RF_11288 have only 25.08% sequence homology but are predicted to have similar core crystal structures with distal helices encompassing putative transmembrane domains (Fig. S6A). Predicted structural homologs of these proteins included putative transcription factors and uncharacterized proteins but also well-characterized cell wall-modifying enzymes such as Csp2A from Streptococcus pneumoniae D39 (PDB 4DE8 [47]), LcpA from Staphylococcus aureus N315 (PDB 6UEX [48]), and TagU from Bacillus subtilis 168 (PDB 6UF6 [48]) (Table S3 and Fig. S6B). This suggests that the OG1RF_10350 and OG1RF_11288 mutants may modify the E. faecalis cell wall, which could affect the ability of these mutants to form biofilms under the conditions we tested.

10.1128/mBio.01011-21.6 FIG S6 Predicted crystal structures for OG1RF_10350, OG1RF_11288, and OG1RF_11456. (A) Phyre2 was used to predict the structures of OG1RF_10350 and OG1RF_11288. Both proteins have predicted transmembrane domains (shown as gray boxes in cartoons on the right). (B) OG1RF_10350 and OG1RF_11288 have predicted structural homology to multiple LCP family wall teichoic acid transferases from Gram-positive bacteria. PDB identifiers for Cps2A, LcpA, and TagU are shown. Lipid substrates for Cps2A and LcpA are represented by black spheres. (C) The putative crystal structure of OG1RF_11456 has predicted structural homology to membrane-bound chemosensors Tsr and Tm14. OG1RF_11456 has one predicted transmembrane domain (shown as a gray box in the cartoon on the right and as black residues in the OG1RF_11456 predicted structure). Tsr residues that undergo methylation are shown as black spheres. Download FIG S6, TIF file, 0.6 MB. Copyright © 2021 Willett et al. 2021 Willett et al. https://creativecommons.org/licenses/by/4.0/ This content is distributed under the terms of the Creative Commons Attribution 4.0 International license.

OG1RF_11456 is annotated as a methyl-accepting chemotaxis receptor, although E. faecalis is nonmotile. Biofilms formed by the OG1RF_11456-Tn mutant contained large multicellular aggregates (Fig. 3C and F, 4C, and 5C). Phyre2 analysis of OG1RF_11456 yielded high-confidence matches to the methylation and signaling domains of Tsr, the membrane-bound serine chemotaxis receptor from E. coli (PDB 1QU7 [49]), and Tm14, a chemoreceptor from Thermotoga maritima (PDB 3G67 [50]) (Table S3). The putative structure of OG1RF_11456 is an extended linear conformation, similar to that of Tsr and Tm14 (Fig. S6C). OG1RF_11456 has a predicted transmembrane domain that best aligns with the Tsr/Tm14 signaling domains, which are cytoplasmic (49, 50). Although the Tsr methylation sites are not conserved in OG1RF_11456, this protein contains multiple glutamine and glutamic acid residues that could be involved in signal transduction. However, additional experiments are needed to confirm whether OG1RF_11456 functions as a signaling protein in E. faecalis and how this relates to the extreme clumping phenotypes observed in OG1RF_11456-Tn biofilms.

Numerous *in vitro* biofilm determinants of OG1RF have also been characterized as virulence factors in models of biofilm-associated infections (5). One such protein is GelE (gelatinase), a secreted metalloprotease regulated by the Fsr quorum sensing system; *gelE*-negative mutants show defects in biofilm formation *in vitro* and are attenuated in animal models (51, 52). Therefore, we tested whether the 43 Tn mutants chosen for 96-well plate biofilm assays could secrete active GelE. Mutants were spotted on agar plates containing 3% gelatin, and colonies were evaluated for the production of an opaque zone indicative of gelatinase activity (52). All mutants except for the *prsA*-Tn (OG1RF_10423-Tn) mutant had gelatinase-positive phenotypes similar to that of OG1RF (Fig. S6). PrsA is a predicted extracellular membrane-bound peptidyl-prolyl *cis-trans* isomerase that is associated with tolerance to salt stress (53) and E. faecalis virulence in Galleria mellonella (53) and is upregulated in a rabbit subdermal abscess model (43), although no specific protein substrates for chaperone or foldase activity have been identified. We suspect that PrsA enhances correct folding of GelE as it transits the membrane during secretion. The cumulative results from this study suggest important roles for several poorly characterized gene products as important modulators of biofilm formation and architecture.

## DISCUSSION

In this study, we cultured a library of E. faecalis OG1RF Tn mutants in CDC biofilm reactors and identified new determinants of biofilm formation using TnSeq. We identified core biofilm determinants in OG1RF by comparing our results to previous studies done using microtiter plate biofilm assays (23, 27). While the endpoint measurement of both experiments is biofilm formation, microtiter plate assays test the ability of a strain to form a biofilm when grown as a monoculture, whereas TnSeq measures the fitness of a community of mutants. As such, it is expected that some mutants behave differently in these assays, and there is value in using TnSeq to study biofilm formation even in species or strains that have been extensively used in microtiter plate experiments. Using the same Tn library to identify biofilm determinants under multiple conditions can allow for categorization of core biofilm determinants and condition-specific accessory determinants. Core biofilm determinants could be promising targets for the development of new antibiofilm or antimicrobial therapeutics.

Experimental bottlenecks can result in stochastic loss of mutant during TnSeq (54). In a previous study of OG1RF growth in CBRs, ∼10^6^ biofilm CFU/disc were recovered after 4 to 6 h of static growth and ∼10^8^ biofilm CFU/disc were recovered after 24 h of total growth (28). We believe that the small size of our Tn libraries coupled with the number of discs used per CBR reduced the effect of bottlenecks in our primary screens. Determining Tn abundance in biofilms is further complicated because both replication of adherent cells and attachment of new cells from planktonic culture can contribute to overall biofilm growth. We identified mutants underrepresented in biofilms by comparison to abundance in planktonic culture instead of the input population. While this comparison allows for identification of biofilm-specific defects as opposed to general growth defects, we may have missed some mutants with biofilm phenotypes by not comparing to the input library.

While this is primarily a comprehensive gene discovery study, we gleaned some mechanistic insights through structure/function predictions that will serve as the basis for future studies. Additionally, disruption of either *prsA* (peptidyl-prolyl isomerase) or *tig* (trigger factor) led to substantial alteration of biofilm morphology. Of the 4 genes encoding proteins with peptidyl-prolyl isomerase (PPIase) domains in OG1RF (*prsA*/EF0685, *tig*/EF0715, OG1RF_11253/EF1534, and OG1RF_12199/EF2898 [53]), only *prsA*-Tn and *tig*-Tn mutants were underrepresented in our biofilm study. Additionally, the *prsA*-Tn mutant had a gelatinase-negative phenotype when grown on gelatin plates, but the *tig*-Tn mutant was gelatinase positive. Determining the substrates of the OG1RF PPIases is crucial for understanding how aberrant protein folding and secretion affect biofilm architecture and growth.

We used two growth media (TSB-D and MM9-YEG) to generate a more comprehensive view of how growth conditions affect E. faecalis biofilms. These results demonstrate that growth medium can significantly influence genetic determinants of biofilm formation, given the number of mutants identified in TSB-D compared to that in MM9-YEG as well as the small overlap of mutants identified in the two media. Additionally, an increase in multicellular chains was observed in TSB-D biofilms compared to those grown in MM9-YEG (see Fig. S2B in the supplemental material; and compare Fig. 4C with Fig. 5C), whereas OG1RF biofilms grown in MM9-YEG for 24 h were thicker than those grown in TSB-D. Glucose availability is a significant difference between TSB-D (no added glucose) and MM9-YEG (0.4% added glucose), although other nutritional differences might affect biofilm formation. This provides a rationale for testing multiple growth conditions during genetic screens and suggests that nutritional availability in different host niches, such as the GI tract compared to wounds or abscesses, could affect determinants of biofilm growth.

Examining temporal biofilm formation also revealed important morphological variations. In general, biofilms cultured for 24 h in MultiRep reactors had a marked decrease in cell chain length compared to biofilms cultured for 4 h. However, multiple factors such as time or fluid flow might influence these architectural changes. Based on our results, extrapolating the influence of biofilm determinants between growth conditions should be done with caution; previously, we found that only a minority of genes identified as biofilm determinants using *in vitro* screens affected virulence in experimental infections involving biofilm growth (34). Additional work is needed to understand how nutrient availability and the temporal nature of biofilm development affect biofilm determinants, biofilm morphology, and matrix composition at different sites of infection or colonization, including niches not associated with a mammalian host.

Validating mutants identified in a primary screen is a major challenge with TnSeq and other high-throughput genetic experiments. Here, we tested biofilm-deficient mutants identified from CBR TnSeq in three subsequent biofilm assays (microtiter plates, submerged Aclar discs, and MultiRep reactors) that represent a trade-off between throughput and similarity to the primary screen. Microtiter plate assays allow simultaneous testing of dozens to hundreds of mutants using small sample volumes, but they are “closed” systems incubated under static conditions without supplementation of fresh growth medium. Despite the dissimilarity of microtiter plates and CBRs, ∼30% of the Tn mutants we tested had defects in biofilm formation in 96-well plates, suggesting that these may be a reasonable platform for secondary screens of large sets of mutants in order to identify those with reproducible phenotypes for subsequent studies. However, this must be balanced against the probability of excluding mutants with CBR-specific (or flow-specific) biofilm-deficient phenotypes. Although submerged Aclar disc assays and MultiRep reactors can more closely mimic the conditions of CBRs, these are more suitable for smaller sets of mutants given the time and resources required to process, quantify, and visualize samples. Fresh growth medium can be provided to cultures grown in MultiRep reactors, enabling the study of biofilms under flow conditions with lower reagent requirements than CBRs and increasing the feasibility of studies in the presence of antibiotics or other compounds.

From the underrepresented Tn mutants identified in biofilm TnSeq, we chose 6 mutants for quantification and visualization of biofilms. Figures 3 and 8 show the biofilm phenotypes of these mutants relative to one another in various assays. Figure S8 (available at Figshare at https://doi.org/10.6084/m9.figshare.14403284) also presents the images from monoculture experiments grouped by mutant, facilitating direct comparison of multiple biofilm phenotypes of the various mutants in different assays. Importantly, quantification of biofilm cells did not correlate with biofilm morphology. Relying on quantitative measurements of biofilm formation to identify differences between strains may obscure important variances in morphology or developmental processes such as biofilm remodeling or cellular exodus (16). Quantification of biofilm and planktonic cells also suggested that the Tn mutants used in cocultures could compete with OG1RF under most conditions. Interestingly, we found that the *prsA*-Tn mutant grew better when cocultured with OG1RF in TSB-D than when grown alone. However, these Tn mutants were originally identified as underrepresented in TnSeq, so perhaps the complexity of the Tn library restricts growth of certain mutants in biofilms.

Multiple genes in the *epa* operon were also underrepresented in biofilm TnSeq. With the exception of *epaQ*, these are all part of the variable region downstream of genes encoding the core rhamnopolysaccharide backbone (55). Modification of the Epa backbone or side chains affects biofilm architecture, antibiotic-associated biofilm formation, and resistance to phage and antibiotics (16, 25, 26, 33, 34, 55, 56). However, our previous work did not identify EpaOX and EpaQ as important for biofilm formation in the absence of antibiotics or cell wall stressors (26, 34). These studies quantified biofilm formation in microtiter plates, so perhaps these *epa* genes are important for biofilm integrity in the presence of shear stress generated in CBRs. Recently, Guerardel et al. proposed that addition of teichoic acid to the rhamnan backbone and anchoring of Epa to the cell wall may be mediated by LCP family proteins (55). OG1RF encodes five LCP family proteins, two of which we identified as important for biofilm formation (OG1RF_10350 and OG1RF_11288). The predicted crystal structures of these proteins have high homology to LCP family wall teichoic acid transferases in other Gram-positive bacteria (46, 47). Interestingly, OG1RF_10350-Tn and OG1RF_11288-Tn biofilms had increased chaining and clumping compared to OG1RF when grown in MM9-YEG, and *epaOX* and *epaQ* mutant strains also form biofilms with altered morphology (16, 34). Additional work is needed to identify the targets and substrates of LCP family proteins in OG1RF and how cell wall integrity and composition are affected in their absence.

Overall, our study identified sets of new and core biofilm determinants for E. faecalis OG1RF and found that disruption of multiple biofilm determinants leads to drastic changes in biofilm morphology during monoculture and coculture. We also identified specific morphological signatures of OG1RF biofilms grown in different media, with biofilms grown in TSB-D containing mostly multicellular chains and biofilms grown in MM9-YEG containing mostly single cells. Many newly identified biofilm determinants are poorly characterized proteins or intergenic regions, suggesting that our understanding of enterococcal biofilm formation under diverse conditions is still incomplete. Additionally, we identified potential roles in production of gelatinase, Epa, and cell wall homeostasis for multiple new biofilm determinants. Taken together, our work shows how E. faecalis biofilm architecture can be modified by growth medium, experimental conditions, and genetic determinants, demonstrating that comparing biofilms across multiple conditions can provide new insights into the process of biofilm formation as well as basic bacterial biology.

## MATERIALS AND METHODS

### Bacterial strains and growth conditions.

Bacterial strains were maintained as freezer stocks at −80°C in 20 to 25% glycerol. Strains were routinely grown in brain heart infusion (BHI) broth for cloning and generating freezer stocks. All strains used in this study are listed in Table S4 in the supplemental material. Overnight cultures were grown in the same medium used for experiments. Antibiotics were used at the following concentrations: chloramphenicol (Cm), 10 μg/ml; erythromycin (Erm), 10 μg/ml (E. faecalis) or 80 μg/ml (E. coli); fusidic acid (FA), 25 μg/ml; tetracycline (Tet), 5 (liquid) or 10 (plates) μg/ml. When required, agar was added to the growth medium at a final concentration of 1% (wt/vol). MM9-YEG (modified M9 growth medium supplemented with yeast extract and glucose) was prepared as previously described (29). BHI and tryptic soy broth without added dextrose (TSB-D) were purchased from BD and prepared according to manufacturer’s instructions. Fusidic acid was purchased from Chem-Impex, and all other antibiotics were purchased from Sigma.

### Cloning and Tn mutant verification.

Nucleotide sequences of primers are listed in Table S3. All restriction enzymes were purchased from New England Biolabs. For construction of the cCF10-inducible *tig* complementation vector, *tig* was amplified from purified OG1RF genomic DNA using *Pfu* Ultra II polymerase (Agilent), digested with BamHI-HF/NheI-HF, and ligated to pCIEtm (23) treated with the same restriction enzymes. The plasmid construct was verified by Sanger sequencing (Eurofins). For generation of constitutive fluorescent protein constructs, P_23_ was excised from pDL278p23 (57) by digestion with EcoRI-HF/BamHI-HF and ligated to pTCV-LacSpec digested with the same restriction enzymes. A fragment encoding promoterless GFP (58) flanked by BamHI and BlpI sites was inserted to create pP_23_::GFP, and the BamHI-SphI fragment from pJ201::187931 was inserted to create pP_23_::tdTomato. The Tn insertions in strains used for submerged Aclar disc and MultiRep reactor experiments were verified by colony PCR using the oligonucleotides listed in Table S4. The Tn insertion adds ∼2.1 kb to the size of the wild-type allele.

### CDC biofilm reactors.

Reactors were assembled as previously described (14, 16, 28) and incubated at 37°C overnight to ensure a lack of contamination. Polycarbonate (BioSurfaces Technologies Corp.) and Aclar (Electron Microscopy Sciences) discs were used as biofilm substrates (at least 12 discs per replicate). Immediately prior to inoculation, single-use Tn library aliquots were removed from storage at −80°C and thawed on ice. Growth medium (either MM9-YEG or TSB-D) was inoculated with 6 × 10^8^ to 2 × 10^9^ CFU. Batch cultures were grown without flow for 4 to 6 h, after which the peristaltic pump (Cole Parmer) was turned on at a flow rate of 8 ml/min for 18 to 20 h (total experiment time, 24 h). In a previous study, this resulted in at least 10^6^ biofilm CFU/disc after 4 to 6 h of static culture and 10^8^ biofilm CFU/disc after 24 h (28), reducing the possibility of a bottleneck restricting growth of the Tn libraries. Two biological replicate reactors were run for each Tn library-growth medium combination.

### DNA isolation, library preparation, and transposon sequencing.

Substrates were removed from the CDC biofilm reactor chamber and processed to remove adherent biofilm cells. Polycarbonate discs were aseptically removed and placed in 6-well plates (4 discs/well) containing 5 ml distilled water and incubated for 5 min at room temperature to remove nonadherent cells. To obtain attached biofilm cells, 12 discs were placed in 50-ml conical tubes containing 30 ml KPBS (potassium phosphate-buffered saline, pH 7.0) with 2 mM EDTA and vortexed in a BenchMixer multitube vortexer (Benchmark Scientific) at 2,000 rpm for 5 min. Biofilms grown on Aclar membranes were rinsed in 50-ml conical tubes with 30 ml KPBS, followed by inversion to remove nonadherent cells. Rinsed Aclar membranes were submerged in 4 ml KPBS with 2 mM EDTA, and biofilms were removed by scraping with a sterile razor blade. Biofilms from multiple substrates from each reactor were pooled in a conical tube, pelleted at 6,371 × *g* for 15 min, and frozen at –80°C until further use. Pellets were resuspended in 180 μl enzymatic lysis buffer (20 mM Tris-HCl [pH 8.0], 2 mM EDTA, 1.2% Triton X-100) with 30 mg/ml lysozyme and 500 U/ml mutanolysin. After 30 min of incubation at 37°C, 25 μl proteinase K and 200 μl buffer AL (DNeasy blood and tissue kit; Qiagen) were added. Tubes were incubated at 55°C for 30 min, after which DNA was extracted using a DNeasy blood and tissue kit by following the manufacturer’s instructions. Samples were submitted to the University of Minnesota Genomics Center for library preparation and sequencing. Sequencing libraries were prepared using the Illumina TruSeq nano library preparation kit as previously described (22). Libraries were sequenced as 125-bp paired-end reads on an Illumina HiSeq 2500 in high-output mode (440M reads total).

Sequencing reads were processed using a published workflow (22). Briefly, reads were trimmed and aligned to the OG1RF genome (NC_017316.1), and Tn insertions at TA sites were quantified. The statistical significance of the relative abundance of Tn reads at each TA site in biofilm compared to planktonic samples was evaluated using a chi-square test and an additional Monte Carlo-based method as an evaluation of the accuracy of the chi-square test. Scripts for all processing steps are publicly available (https://github.com/dunnylabumn/Ef_OG1RF_TnSeq). Output files were filtered for nucleotide positions of Tn mutants known to be present in the library based on previous sequencing (22). Log_2_ fold changes were calculated from relative Tn abundances in biofilm compared to planktonic samples. Statistical significance was defined as a *P* of <0.05 and a Monte Carlo simulation value of 1.119552, the lowest value obtained in these calculations. Venn diagrams showing overlap between data sets were generated with the VennDiagram package for R (59).

Tn mutants used for additional experiments were obtained from frozen library stock plates and grown on BHI/FA agar plates. Single colonies were picked and patched onto BHI/Erm to ensure loss of the plasmids used in Tn mutagenesis and BHI/Cm to confirm functionality of the Cm resistance gene located in the Tn. Single colonies were picked from BHI/Cm plates and grown in BHI/Cm/FA to generate freezer stocks. Tn insertions were verified by colony PCR using primers flanking the gene of interest (Table S3). The Tn adds ∼2.1 kb to the size of the parental allele (27, 60).

### SEM.

Biofilms were removed from the CBRs and rinsed with KPBS three times and then processed for scanning electron microscopy (SEM) using the cationic dye stabilization methods described previously (14–16, 34). Briefly, biofilms were subjected to primary fixation in sodium cacodylate buffer containing methanol-free EM-grade formaldehyde (2%), glutaraldehyde (2%), sucrose (4%), and Alcian blue 8GX (0.15%) overnight. Discs were then rinsed three times with sodium cacodylate buffer and subjected to secondary fixation in sodium cacodylate buffer containing 1% osmium tetroxide and 1.5% potassium ferrocyanide for 1 h. Fixed samples were rinsed three times with sodium cacodylate buffer and chemically dried using a graded ethanol series, processed in a CO_2_-based critical-point dryer (Tousimis, Rockville, MD), and sputter coated with ∼2 nm iridium (EM ACE600; Leica, Buffalo Grove, IL). Imaging was done using a Hitachi SU8230 field emission instrument at 0.8 kV using the low-angle backscatter and secondary electron detectors.

### Biofilm assays.

Ninety-six-well plate biofilm assays were carried out as described previously (23, 26, 30). Overnight cultures for complementation assays were grown with 5 μg/ml tetracycline and 25 ng/ml cCF10, and experiments were performed in the indicated growth medium supplemented with 25 ng/ml cCF10. Briefly, overnight cultures were diluted 1:100 in the appropriate growth medium, and 100 μl was added to a 96-well plate (Corning 3935). For the secondary screens using 43 Tn mutants, two technical replicates were performed for each strain. For complementation assays, three technical replicates were performed for each strain. For all experiments, values shown are the results of three independent biological replicates. Plates were incubated in a humidified plastic container at 37°C for the indicated length of time. Cell growth was measured in a Biotek Synergy HT plate reader as the absorbance at 600 nm (*A*_600_). Plates were gently washed three times with ultrapure water using a BioTek plate washer, dried in a biosafety cabinet or on a lab bench overnight, and stained with 100 μl 0.1% safranin (Sigma). Stained plates were washed three times and dried. The *A*_450_ was measured to quantify safranin-stained biofilm biomass. Biofilm production was evaluated as the ratio of stained biofilm biomass to overall growth (*A*_450_/*A*_600_), and values were normalized to the biofilm production of OG1RF.

For submerged Aclar biofilm assays, overnight cultures were adjusted to 10^7^ CFU/ml in the appropriate growth medium, and 1 ml was added to 1 well of a 24-well plate (Costar 3524) with a 5-mm Aclar disc. Plates were incubated at 37°C in a plastic container on a tabletop shaker (Thermo Scientific MaxQ 2000) at 100 rpm. After 6 h, planktonic cells were transferred to microcentrifuge tubes. Aclar discs were washed by gentle shaking in KPBS and transferred to microcentrifuge tubes with 1 ml KPBS (1 Aclar disc/tube). Tubes with planktonic cultures and Aclar discs were vortexed at 2,500 rpm for 5 min in a BenchMixer multi-tube vortexer (Benchmark Scientific) and then diluted (10-fold serial dilutions) in KPBS and plated on BHI/FA medium to enumerate colonies. For coculture experiments, diluted cultures were plated on BHI/FA (total CFU counts) and BHI/Cm plates (Tn mutant CFU counts). CFU/ml values for OG1RF in coculture were obtained by subtracting the CFU/ml counts from BHI/Cm plates from the CFU/ml counts from BHI/FA plates. At least three biological replicates (each with two technical replicates) were performed for all strains.

MultiRep biofilm reactors (Stratix Labs, Maple Grove, MN) were loaded with 5-mm Aclar discs (6 Aclar discs per channel). Influx (MasterFlex HV-96117-13) and efflux (MasterFlex EW-06424-16) tubing was attached to each channel and capped with foil prior to autoclaving. The 10% growth medium was autoclaved in a separate bottle with sterile connecting tubing and attached to the influx reactor tubing immediately prior to inoculation. Overnight cultures were diluted to 1 × 10^7^ CFU/ml, and 4 ml was added to each channel (1 channel per strain). The reactor was sealed by placing 2 silicon sheets in the lid and clamping the lid on the reactor using Irwin Quick-Grip ratcheting bar clamps. The influx tubing was connected to peristaltic pumps (MasterFlex 77202-60), and the efflux tubing was placed horizontally over waste containers. Reactors were kept at 37°C with static incubation for 4 h, after which the pumps were turned on at a flow rate of 0.1 ml/min for 20 h. For disassembly and sample processing, the reactor lids were removed, and 2 ml planktonic culture was transferred to microcentrifuge tubes. Aclar discs were removed and rinsed in KPBS and then placed in microcentrifuge tubes with 1 ml KPBS. Tubes with planktonic cultures or Aclar discs were vortexed and diluted, and the numbers of CFU were enumerated as described above.

### Fluorescence microscopy.

For all experiments, Aclar discs (2 per strain) were rinsed 3 times in KPBS and stained for 15 min in Hanks’ balanced salt solution with CaCl_2_ and MgCl_2_ (Gibco) and 5 μg/ml Hoechst 33342 (Molecular Probes) with gentle agitation. After staining, Aclar discs were washed 3 times in fresh KPBS, transferred to a 48-well plate (Costar 3548) with 1 ml 10% buffered formalin (Fisher Scientific) with gentle agitation, and shielded from light for 12 to 16 h. After fixing, Aclar discs were washed in KPBS and mounted on a Superfrost Plus microscope slide (Fisher Scientific) in a 0.24-mm double-sided adhesive Secure-Seal spacer (Grace BioLabs) with a 7-mm hole punched to accommodate the Aclar disc. Aclar discs were covered with 7 μl ProLong glass antifade mountant (Invitrogen) and a Gold Seal cover slip (no. 1.5; Fisher Scientific). Slides were cured at room temperature shielded from light for 4 to 8 h and stored at 4°C until imaging.

### Microscopy and image processing.

All images were acquired on a Zeiss Axio Imager M1 widefield microscope with a Plan-APO 20× (0.8 numerical aperture [NA]) using an X-Cite 120 metal halide light source (EXFO, Inc.) illuminating 365-nm, 470-nm, and 550-nm excitation filters for Hoechst 33342, GFP, and tdTomato, respectively. Images were captured using the Zeiss AxioCam 503 mono microscope camera and Zen imaging software (v2.1; Zeiss). For each Aclar disc, two independent images were obtained, yielding four images per sample from which a final representative image was chosen. Representative images were processed using the Fiji ImageJ package (version 1.48; NIH) and subjected to background subtraction with a rolling-ball radius of 50 pixels using the internal ImageJ function as well as uniformly applied brightness and contrast adjustments of the entire image prior to cropping (61). For biofilm coculture images, the Hoechst, GFP, and tdTomato images were false colored cyan, yellow, and magenta, respectively, using Fiji. For coculture images, tdTomato (OG1RF) and GFP (Tn mutant) maximum intensity projections (MIPs) were processed independently and merged. Images were cropped to 500 by 500 pixels using GIMP (v 2.0) and exported as PNG files. The GFP (mutant) and tdTomato (OG1RF) MIPs were processed independently and merged.

Biofilm thickness and distribution were analyzed using Comstat2. Cells were imaged using an Axio Observer.Z1 confocal microscope equipped with an LSM 800-based Airyscan system in normal confocal mode (Zeiss). Confocal images were acquired with a 20 by 0.8 NA objective and 405-nm lasers for excitation of Hoechst 33342 stain. For image analysis, two representative z stacks were taken per Aclar disc with a 1-μm interval. Each experiment used three independent biological replicates with at least 2 Aclar discs in each. The maximum thickness of the biofilms was determined from the Hoechst channel using the Comstat2.1 plugin for ImageJ (41, 62). All image processing adheres to the standards outlined by Rossner and Yamada (63).

### Gelatinase assays.

Overnight cultures were grown in the respective growth medium and spotted onto TSB-D agar plates supplemented with 3% (wt/vol) gelatin. Plates were incubated overnight at 37°C and then moved to 4°C for 1 to 3 h prior to imaging. Plate photos were obtained using a ProteinSimple (Cell Biosciences) FluorChem FC3 imager. Strains were considered gelatinase positive if they developed a halo around colony growth and gelatinase negative if no halo was present.

### Bioinformatic analysis.

Functional annotations of proteins were obtained from KEGG and NCBI. Protein sequences were obtained from NCBI and used as input for Phyre2 in intensive mode (45). Transmembrane predictions were obtained with TMHMM (64). Additional protein structure files were downloaded from the Protein Data Bank (PDB), and structures were rendered in PyMOL 2.1 (65).

### Statistical analysis.

All statistical analysis was carried out using GraphPad Prism (version 9.0.1). Statistical tests and significance are described in the figure legends. Corrections for multiple comparisons were performed using the test recommended by GraphPad.

### Data availability.

All sequencing data generated during this study have been deposited in the NCBI GEO database (accession number GSE171419). Figure S8 is available at Figshare (https://doi.org/10.6084/m9.figshare.14403284).

10.1128/mBio.01011-21.7 FIG S7 Gelatinase activity of Tn mutants chosen for microtiter plate biofilm assays. Overnight cultures grown in TSB-D were spotted onto a TSB-D agar plate supplemented with 3% gelatin. After overnight growth, plates were refrigerated until the zone surrounding the colonies was visible. Three biological replicates were performed, and a representative image is shown. Download FIG S7, TIF file, 0.9 MB. Copyright © 2021 Willett et al. 2021 Willett et al. https://creativecommons.org/licenses/by/4.0/ This content is distributed under the terms of the Creative Commons Attribution 4.0 International license.
